# Pangenome analysis provides insights into the genetic diversity, metabolic versatility, and evolution of the genus *Flavobacterium*

**DOI:** 10.1128/spectrum.01003-23

**Published:** 2023-08-18

**Authors:** Minji Kim, In-Tae Cha, Ki-Eun Lee, Meng Li, Soo-Je Park

**Affiliations:** 1 Department of Biology, Jeju National University, Jeju, South Korea; 2 Microorganism Resources Division, National Institute of Biological Resources, Incheon, South Korea; 3 Archaeal Biology Center, Institute for Advanced Study, Shenzhen University, Shenzhen, China; 4 Shenzhen Key Laboratory of Marine Microbiome Engineering, Institute for Advanced Study, Shenzhen University, Shenzhen, China; State Key Laboratory of Microbial Resources, Institute of Microbiology, Chinese Academy of Sciences, Beijing, China

**Keywords:** *Flavobacterium*, pangenome, comparative genomics, evolution, proteorhodopsin, carbohydrate-active enzymes

## Abstract

Members of the genus *Flavobacterium* are widely distributed and produce various polysaccharide-degrading enzymes. Many species in the genus have been isolated and characterized. However, few studies have focused on marine isolates or fish pathogens, and in-depth genomic analyses, particularly comparative analyses of isolates from different habitat types, are lacking. Here, we isolated 20 strains of the genus from various environments in South Korea and sequenced their full-length genomes. Combined with published sequence data, we examined genomic traits, evolution, environmental adaptation, and putative metabolic functions in total 187 genomes of isolated species in *Flavobacterium* categorized as marine, host-associated, and terrestrial including freshwater. A pangenome analysis revealed a correlation between genome size and coding or noncoding density. *Flavobacterium* spp. had high levels of diversity, allowing for novel gene repertories via recombination events. Defense-related genes only accounted for approximately 3% of predicted genes in all *Flavobacterium* genomes. While genes involved in metabolic pathways did not differ with respect to isolation source, there was substantial variation in genomic traits; in particular, the abundances of tRNAs and rRNAs were higher in the host-associdated group than in other groups. One genome in the host-associated group contained a *Microviridae* prophage closely related to an enterobacteria phage. The proteorhodopsin gene was only identified in four terrestrial strains isolated for this study. Furthermore, recombination events clearly influenced genomic diversity and may contribute to the response to environmental stress. These findings shed light on the high genetic variation in *Flavobacterium* and functional roles in diverse ecosystems as a result of their metabolic versatility.

**IMPORTANCE:**

The genus *Flavobacterium* is a diverse group of bacteria that are found in a variety of environments. While most species of this genus are harmless and utilize organic substrates such as proteins and polysaccharides, some members may play a significant role in the cycling for organic substances within their environments. Nevertheless, little is known about the genomic dynamics and/or metabolic capacity of *Flavobacterium*. Here, we found that *Flavobacterium* species may have an open pangenome, containing a variety of diverse and novel gene repertoires. Intriguingly, we discovered that one genome (classified into host-associated group) contained a *Microviridae* prophage closely related to that of enterobacteria. Proteorhodopsin may be expressed under conditions of light or oxygen pressure in some strains isolated for this study. Our findings significantly contribute to the understanding of the members of the genus *Flavobacterium* diversity exploration and will provide a framework for the way for future ecological characterizations.

## INTRODUCTION

The genus *Flavobacterium* in the phylum Bacteroidota (formerly Bacteroidetes) (1) consists of 374 species (https://lpsn.dsmz.de/genus/flavobacterium, accessed on November 2022) isolated from various environments, including the rhizosphere, freshwater, sediment, and sea ice (2). However, correct names for only 270 strains have been validly published. *Flavobacterium* species are aerobic, Gram-negative bacteria that have a rod-shaped cell morphology and glide on surfaces. They can adapt to various environmental conditions, such as a wide range of salt (i.e., NaCl) concentrations or temperatures (2 – 4). The genus includes opportunistic pathogens (2, 5). Studies of pathogenicity have mostly focused on *Flavobacterium* isolated from fish (e.g., juvenile rainbow trout), spoilage foods, or clinical species (5 – 12). Nevertheless, most species of the genus *Flavobacterium* are harmless and utilize organic substrates (e.g., proteins and polysaccharides) in their habitats. Some *Flavobacterium* spp. have a critical role in the cycling of organic matter (uptake, degradation, and decomposition) in diverse environments, with implications for bioremediation (13 – 15). Some strains play crucial roles in plant growth and protection (16 – 18).

Primary research on the genus has largely focused on the identification and characterization of novel species from marine or terrestrial environments despite their putative ecological importance in diverse habitats. Habitat diversity can result from genomic integration of advantageous genes for adaptation to heterogenous ecological niches by horizontal gene transfer (HGT) (19). In addition, variation in genomic contents and genetic redundancy is closely related evolutionary adaptation (20, 21). The integration of exogenous DNAs and deletion of unessential genetic information by mobile genetic elements (e.g., prophages or transposons) are crucial processes for microbial survival under environmental stresses and microbial evolution (22 – 24).

Genomic traits have been studied for a limited number of pathogenic species in the genus *Flavobacterium*. However, an evolutionary, metabolic versatility or genomic dynamic investigation of the genus is lacking using pangenome analysis. Therefore, in this study, we isolated *Flavobacterium* strains from various samples in South Korea and fully sequenced their genomes. We then evaluated genomics traits with the publicly available *Flavobacterium* genomes by a pangenome approach. We grouped genomes based on the isolation sources [i.e., host-associated, terrestrial including freshwater and marine (25)] for comparative analyses of genomic properties and putative metabolic features including photoheterotrophs (26). This analysis provides new information on genomic diversity in the genus and contributes to our understanding of its adaptation to diverse habitats.

## RESULTS AND DISCUSSION

### General genomic features

Twenty complete genomes of strains belonging to the genus *Flavobacterium* were sequenced, assembled, and annotated. We detected a single plasmid in the genome of strain N1816 and two plasmids in strain N2155. Excluding plasmids, genome completeness and contamination were 97.8%–99.7% and 0.0%–2.4% (Table S1), respectively, for the closed genome sequences. These estimates were acceptable based on previously established thresholds of >90% completeness and <5% contamination (27). Metagenome-assembled genomes were not included in analyses owing to the potential bias caused by a lower completeness and high contamination. We focused on the genomes of purified strains belonging to the genus *Flavobacterium*. We also included publicly available genomes from representative *Flavobacterium* isolates in GTDB (*n* = 167) (Table S2). For further analyses, the *Flavobacterium* genomes (*n* = 187) were separated according to isolation sites: terrestrial including freshwater (*n* = 135), host-associated (e.g., fecal samples and tissues of diseased trout or salmon; *n* = 34), and marine (*n* = 18).

The characteristics of genomes (e.g., genome size, GC content, number of CDSs, and coding density) are strongly correlated with lifestyles or ecological and metabolic niches (28 – 30). The average length and GC content were 4.00 Mbp (range 2.33–6.10 Mbp) and 35.06% (29.38%–50.93%), respectively (Table S3 and Fig. S1). The length and mean GC contents were highest for *Flavobacterium johnsoniae* UW101 (GCF_000016645) and strain N1718, respectively. These two strains were isolated from terrestrial habitats (soil and lake water). In particular, *F. johnsoniae* exhibits polysaccharide degradation (including chitin) and novel biochemical features, supported by comparative genomics (31). *Flavobacterium dinanensis* UR159 (formerly *Avrilella dinanensis*, GCF_002807015) isolated from human blood and strain N501239 isolated from tidal flat sediments had the lowest genome length and GC content, respectively. The long genome length suggests that most *Flavobacterium* spp. are free living (29, 32). We did not detect significant differences in mean GC contents with respect to habitat type although the GC% differed between some genomes of terrestrial isolates (avg. 35.42%, range 30.02%–50.93%) and other groups (avg. 34.20%, 30.89%–39.77% in host-associated; avg. 33.94%, 29.38%–37.71% in marine) (Table S3).

The number of total CDSs (range 2,205–5,156, avg. 3,448) was positively correlated (Pearson’s correlation *r* ^2^ = 0.98, *r* = 0.990, and *P* < 0.0001) with genome length (bp) (Fig. S2a). The coding density in each genome was 85.16%–93.08% (avg. 89.32%) (Fig. S2b). Significant differences in coding density were found between habitats (Kruskal–Wallis test, *P* = 0.0235), particularly between terrestrial and host-associated groups (*post-hoc* Dunn’s test, *P* = 0.0191), as supported by estimation statistics (i.e., difference between means, Welch’s *t-*test*, P* = 0.0032) (Fig. S2c). In addition, we detected significant correlations between genome size and both coding (*r*_*s*_ = −0.5848 and *P* < 0.0001 calculated by Spearman rank correlation coefficient) and noncoding (*r*_*s*_ = 0.5848 and *P* < 0.0001) densities. Interestingly, the correlation between genome size and coding density was comparable to that observed in Archaea (33). This correlation is consistent with positive selection and a rapid rate of evolution in members of the genus *Flavobacterium* (28, 34). Additionally, this analysis suggests that adaptation in *Flavobacterium* spp. was mediated by weak genome expansion (i.e., DNA gain) and not through host restriction (35).

### Phylogenomic and pangenome analyses

**Fig 1:**
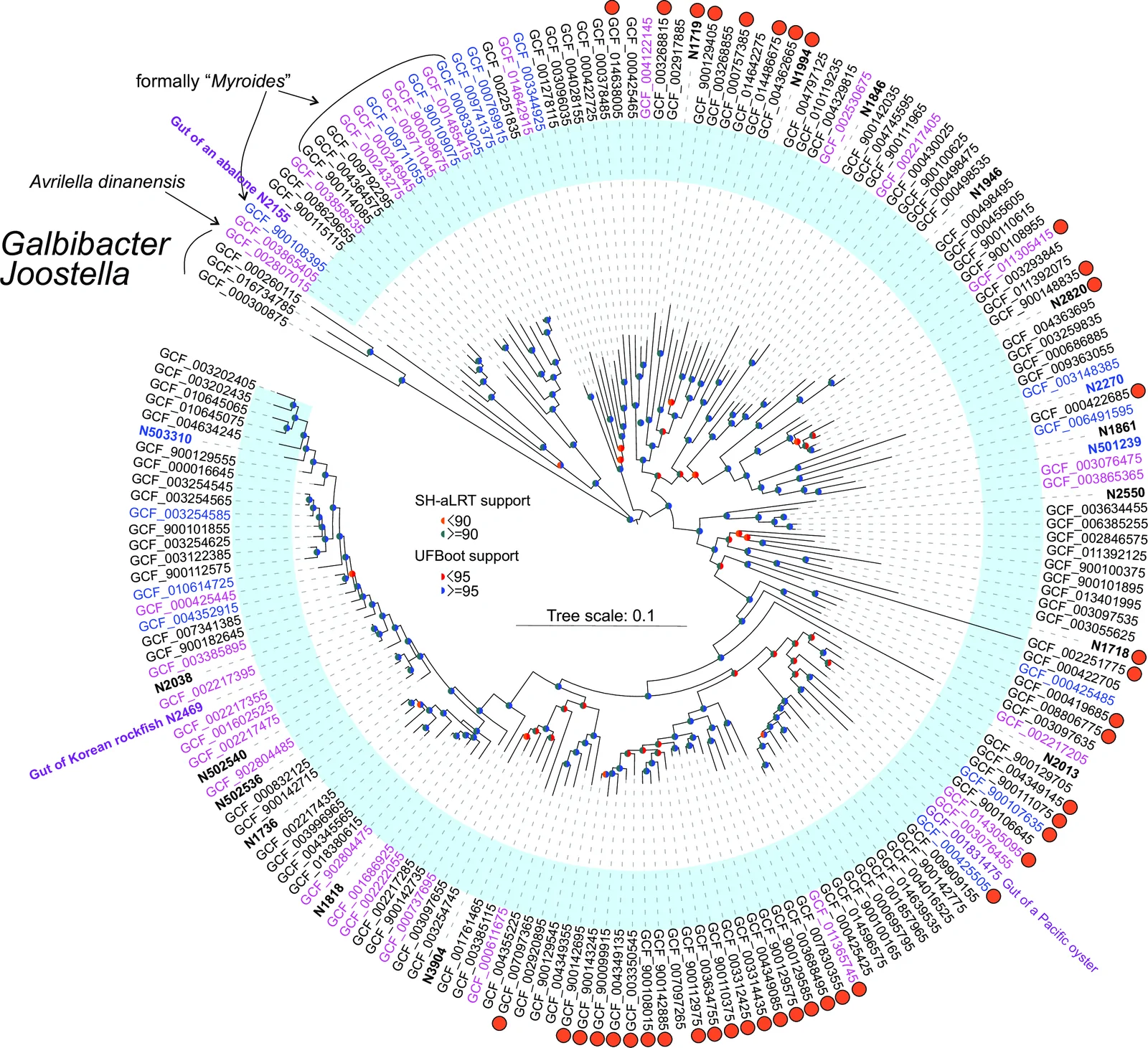
Phylogenomic tree estimated by maximum likelihood inferred from genomes of the genus *Flavobacterium,* including newly isolated strains. The genomes of the isolated strains are highlighted in boldface. Genomes harboring putative proteorhodopsin (PR) sequences are indicated by closed orange circles. The strength of support for internal nodes was estimated by SH-aLRT and ultraboost replicates, and values are shown as colored half-circles (see the legend). The phylogenomic tree was generated with the best-fit substitution model, GTR + F + I + I + R8. The sources (i.e., habitat) of isolates are denoted by black (terrestrial), magenta (gut, stool, and specimens from terrestrial animal hosts), purple (gut of marine animals), and blue (marine). Scale bar represents 0.1 substitutions per site.

Analyses of completely or partially sequenced genomes of purified microbial cells as well as metagenome-assembled genomes for DNA extracted from various environments have expanded our understanding of the metabolic and ecological roles of (novel) micro-organisms. Simultaneously, these data provide a basis for revised phylogenetic analyses. We investigated genomes for the genera *Avrilella* (*n* = 1) and *Myroides* (*n* = 12). The genus *Myroides* has been assigned the *Flavobacterium* clade (2). Several species in the two genera have been isolated from clinical specimens (2, 36). Based on a comparative genome analysis, *Avrilella* and *Myroides* were incorporated into the genus *Flavobacterium* (https://gtdb.ecogenomic.org/), consistent with our phylogenomic analysis, with high values for SH-aLRT and UFBoot (Fig. 1). We did not detect correlations between the phylogenomic position and source of isolation or geological habitat. These results suggested that *Flavobacterium* spp. have high genomic relatedness across ecological niches.

In analyses of genome-wide relatedness, average nucleotide identity (ANI) and average amino acid identity (AAI) values were 64.81%–97.56% (avg. 71.46%) and 56.84%–97.95% (avg. 66.92%), respectively (Fig. S3). Based on these results, we found that four strains isolated in this study might belong to the same species based on the standard cut-off value (>95%) for species delineation based on pairwise comparisons (37): strain N2469 and *Flavobacterium plurextorum* (GCF_002217395), N2550 and *Flavobacterium endophyticum* (GCF_003634455), N1818 and *Flavobacterium bizetiae* (GCF_902804475), and N502540 and *Flavobacterium tructae* (GCF_002217475). Except for these 4 genomes, the other 16 strains isolated in this study might represent novel species within the genus *Flavobacterium*, with lower relatedness (<74.0% and <70.3% for ANI and AAI, respectively) than the proposed species cut-off values. The low and variable estimates of relatedness prompted us to perform additional pangenome analyses.

For the pangenome analysis, 484,043 genes were evaluated using Roary. These genes were assigned to 11 Core (including soft core, ≥95%), 779 Shell (<95%), and 483,253 Cloud (<15%) gene subsets (Fig. S4). The majority genes in the genomes of *Flavobacterium* were classified as accessory (i.e., noncore) genes, suggesting that the genomes are highly plastic. The number of core genes decreased dramatically (Fig. S4a) with the addition of each genome, whereas the number of genes in the pangenome increased (Fig. S4b). To verify this result, genomes of 20 strains isolated in this study were analyzed using Anvio (Fig. S5). In accordance with the results from the analysis of 187 genomes, the majority of genes in 20 genoms analyzed with Anivo were classified as an accessory group. In addition, our results were comparable to those of a previous study of only 27 *Flavobacterium* genomes (38).

Taken together, *Flavobacterium* species might have an open pangenome, harboring diverse, and novel gene repertoires. However, this result is in contrast with the observation of weak genome expansion.

### Potential metabolic characterization

To compare metabolic profiles across isolation sources, predicted genes were searched against the KEGG and Clusters of Orthologous Group (COG) databases (Fig. S6a). Genes for central carbohydrate metabolism (i.e., the Embden-Meyerhof pathway, pyruvate oxidation, tricarboxylic acid [TCA] cycle, gluconeogenesis, pentose phosphate pathway, and Entner-Doudoroff pathway) and electron transport chain were identified in all genomes (see additional data at https://doi.org/10.6084/m9.figshare.22109381.v1). However, we did not detect apparent differences among isolation sources. The results indicate that out of all the COG classes, only two COG classes (V, defense mechanisms; U, intracellular trafficking, secretion, and vesicular transport) were significantly greater enrichment in the marine and host-associated groups compared to the terrestrial group (Kruskal–Wallis test, *P* = 0.0289 and 0.0001, respectively). However, significant differences were only observed between the terrestrial and host-associated groups for COG V (*post-hoc* Dunn’s test, *P* = 0.0466), which was supported by estimation statistics (i.e., difference between means, Welch’s *t-*test*, P* = 0.0124). For COG U, marine and host-associated groups were significantly different (*post-hoc* Dunn’s test *P* = 0.0190 and 0.0008, respectively), supported by estimation statistics (i.e., difference between means, Welch’s *t-*test*, P* = 0.0193 and 0.0018, respectively) (Fig. S6b).

Interestingly, two key enzymes (isocitrate lyase and malate synthase) in the glyoxylate cycle (an anabolic pathway) and an anaplerotic variant of the TCA cycle were found in some genomes (39). In addition, genes [*pccAB* for propionyl-CoA carboxylase, *epi* for (methyl)ethylmalonyl-CoA epimerase, and *scpA* for methylmalonyl-CoA mutase; propionyl-CoA converts to succinyl-CoA] involved in the ethylmalonyl-CoA (EMC) pathway were found in some genomes analyzed in this study. The EMC pathway contributes to central carbon in numerous Alphaproteobacteria and Actinobacteria (40). Although this pathway was incomplete in the genomes of the genus *Flavobacterium*, it is possible that intermediates are supported by other pathways, such as fatty acid metabolism. These findings suggested that some members of the genus *Flavobacterium* have an ecological advantage (i.e., the ability to utilize C_1_ and C_2_ compounds) over competitors in simple sugar-limited environments (41, 42) or have higher putative metabolic flexibility (43) than was previously observed based on physiological characterization.

We identified genes for butyryl-CoA dehydrogenase (Bcd) and electron transfer flavoprotein (EtfAB) in all genomes. The Bcd/Eft complex for energy conservation is found in a few bacterial strains in the genera *Clostridium*, *Acidaminococcus*, and *Megasphaera* of the phylum Bacillota (formerly Firmicutes) (44 – 46). This is the first evidence for these genes in *Flavobacterium* and suggests that members of this genus have high metabolic versatility.

### Carbohydrate metabolism estimated by carbohydrate-active enzymes

Terrestrial *Flavobacterium* spp. have niche adaptation via plant glycan metabolism, including genes for rhamnogalacturonan utilization (47). Recently, Gavriilidou et al. (48) detected substantial variation in genes related to carbohydrate metabolism in the family Flavobacteriaceae, distributed in marine environments and associated with marine (in)vertebrates and algae, based on a comparative genomic analysis. Furthermore, Gavriilidou et al. (48) showed that marine taxa in the family Flavobacteriaceae possess more carbohydrate-active enzymes (CAZymes) per Mbp than corresponding estimates in Cyanobacteria and Proteobacteria. We cannot exclude the importance of carbohydrate metabolism in marine *Flavobacterium* spp. In fact, many studies have investigated the algal polysaccharide-processing abilities of marine heterotrophic bacteria (49). Mann et al. (50) claimed that the marine flavobacterium *Formosa agariphila* degrades high-molecular-weight particulate organic matter (e.g., polysaccharides) provided by corals and algae. These results suggest that marine-associated members of the genus *Flavobacterium* could contribute to organic carbon recycling in their ecological niche (51) or to the morphological development of marine macroalgae (52).

To evaluate differences in carbohydrate degradation and/or transformation ability among isolation sites, the CAZy repertoire was evaluated based on the number of CAZymes (predicted as described in the Materials and Methods) per Mbp (sequence length) (Table S4). We detected slight differences in the number of genes in the CAZy repertoire (Kruskal–Wallis test, *P* = 0.0020). In particular, the significant differences were estimated between terrestrial *Flavobacterium* genomes (avg. 55.78) and marine genomes (avg. 45.64) (*post hoc* Dunn’s test, *P* = 0.0158) and host-associated genomes (avg. 46.34) (*post-hoc* Dunn’s test, *P* = 0.0092). However, we did not detect significant differences between genomes belonging to the marine and host-associated genomes (*post-hoc* Dunn’s test, *P* > 0.9999). In addition, we evaluated the number of CAZymes commonly predicted by three methods (HMMER, Diamond, and Hotpep), revealing significant variation across (isolated) habitats (Kruskal–Wallis test, *P* = 0.0483), with no significant differences between each site. We then compared only the number of CAZymes predicted by at least one method relative to the total number of predicted genes, showing lower values for terrestrial *Flavobacterium* (avg. 17.17) than for host-associated and marine groups (21.26 and 21.92, respectively). However, we observed a statistically significant difference (Kruskal–Wallis test, *P* = 0.0019), particularly, between terrestrial and marine (*post-hoc* Dunn’s test *P* = 0.0198) and host-associated and terrestrial *Flavobacterium* genomes (*post-hoc* Dunn’s test *P* = 0.0239) (Fig. S7). These findings were supported by the estimation statistics (95% confidence interval, Welch’s correction) of *P* = 0.0160 and *P* = 0.0028, respectively (Fig. S7). Taken together, these results suggest that genes encoding CAZymes in genomes of terrestrial *Flavobacterium* might be densely distributed even though the number of the CAZymes was slightly lower than those in the other clades.

To evaluate adaptation, we focused on rhamnogalacturonan (GH78 and 106), pectin (GH28), agar (GH50), and fucoidan (GH29, GH95, 107, and 168) gene families in all genomes (16, 48, 50, 51, 53). No significant differences were observed in the ratio of the selected GH families among the groups, as estimated per genome count. Additionally, the abundance of GH28 (polygalacturonases for pectin hydrolysis) was higher than that of other GH families (Table S4). No GH107 (*endo*-fucosidase for fucan hydrolysis in brown algae) (54) was identified in the genomes, and only a single gene encoding GH168 (in marine invertebrates) (55) was found in the genome (GCF_010645065) of *Flavobacterium fluviatile* isolated from freshwater. Kolton et al. suggested that terrestrial *Flavobacterium* clades have niche specificity for plant-related carbohydrate (i.e., plant hemicelluloses) metabolism via GH78 and GH106 families (16). However, the abundances of the GH 78, 29, and 33 families were similar in terrestrial and marine groups in this study. It is possible that this can be explained by the small number of genomes analyzed in the previous study (i.e., reference 15) (47).

Two GH families (GH29 [fucosidase] and GH50 [beta-agarase]) were slightly more abundant in host-associated *Flavobacterium* genomes than in genomes of other clades (Table S4). In fact, studies of GH29 have mainly focused on terrestrial clades, with only a few reports of the family in marine organisms. This can be explained by the distinct compositions of glycans, such as those for algal cell walls (i.e., fucoidan). It is not clear why GH29 fucosidase genes were more abundant in host-associated genomes than in the other groups. In addition, little is known about the functions of GH29 in the host-associated clade. It is possible that fucoidan functions as a potent inhibitor of cell-to-cell interactions (i.e., smooth cell adhesion) (56).

By a CAZy analysis, we identified four genes related to GH34 (viral sialidases [i.e., neuramidase]) in the genome (GCF_014642275) of *Flavobacterium lutivivi* isolated from activated sludge (57). However, only two genes (IEW38_RS12870||NZ_BMIM01000028 and IEW38_RS12875||NZ_BMIM01000029) were predicted as neuramidase genes (e.g., by blastp) belonging to influenza virus A H9N2 (with an amino acid identity [AAI] and length coverage of 91.202% and 100%, respectively) and influenza virus A H5N6 (91.503% and 97.660%, respectively), respectively, estimated by FluSurver, assessed in November 2022 (https://flusurver.bii.a-star.edu.sg/). In addition, we found the other neuramidase sequence (WP_188959898.1, influenza virus A H9N2, 90.987% and 100%, respectively) in the genome of *Siccirubricoccus deserti* isolated from a desert sand sample (58). Based on the CAZy classification, sialidases are generally classified into four families: GH33, GH34, GH58, and GH3. Additionally, the sialidase family GH156 was recently established via a functional metagenome approach (59). GH33 is a family of well-known nonviral sialidases and has been detected in humans, whereas GH34 is exclusively known as a family of viral sialidases from influenza virus A and B types (60). In this study, GH33 was identified in all genomes (Table S4). To the best of our knowledge, there is no evidence that sialidases belonging to GH34 are encoded in the two above-mentioned bacterial genomes. It is possible that the genes were obtained by HGT through bacteriophages. However, this hypothesis is not supported by our observation that all *Flavobacterium* genomes lack genes for the GH58 family (61).

### Environmental stress or adaptation

Prokaryotes harbor genes for phage defense systems. However, the number of defense systems depends on the genome size and not on the lifestyle of micro-organisms (62). *Flavobacterium* spp. might harbor a great number of genes involved in phage defense. The number of defense system-related genes in all genomes ranged from 61 to 182 (avg. 103.12) (Table S3) and was correlated with the size of the *Flavobacterium* spp. genome (*R* ^2^ = 0.741). The average percentages of total predicted genes classified as defense-related for the terrestrial, host-associated, and marine groups were 2.94%, 3.13%, and 3.06%, respectively. The proportion of defense-related genes differed slightly between the terrestrial and host-associated groups (*post-hoc* Dunn’s test, *P* = 0.0820). These values were comparable to those for pathogenic or intracellular parasitic genera, such as *Rickettsia*, *Francisella*, *Neisseria*, and *Yersinia* (62). In addition, the percentages of genes assigned to the COG V (defense mechanism) for the terrestrial, host-associated, and marine groups were estimated to be 1.76%, 1.94%, and 1.80%, respectively (see Fig. S6). Taken together, these results indicate that representative defense system genes in the bacterial genome are related to environmental conditions.

Most members of the genus *Flavobacterium* have been isolated and characterized in nonmarine environments (e.g., soil and freshwater) (2). Species in the genus have adapted to diverse habitats, without substantial genomic diversity. This suggests that members of the genus have unique strategies for responding to environmental stresses (e.g., temperature and nutrient availability) in their habitats. Adaptation is associated with the numbers of transfer RNAs (tRNAs) (63 – 65) or ribosomal RNAs (rRNAs) (66 – 68) in bacteria. We found 32–104 tRNA genes (avg. 49.82) and 0–19 rRNA genes (avg. 6.75) (Table S3). No rRNA genes were identified in the genome of *Flavobacterium foetidum* (GCF_004634245). The relative abundance of tRNA and rRNA genes was highest in the host-associated group (Fig. S8). In addition, there were no significant differences in the proportion of rRNA and tRNA genes among total genes among groups (Kruskal–Wallis test, *P* = 0.2873 and 0.0990, respectively). However, based on the number of the tRNA and rRNA genes, significant difference was only found in the tRNA gene count with respect to isolation sources (Kruskal–Wallis test, *P* < 0.0001). In particular, the tRNA gene count differed significantly between the host-associated and terrestrial groups (*post-hoc* Dunn’s test, *P* < 0.0001) and supported estimation statistics (i.e., difference between means, *P* = 0.0002) (Fig. S8). While host-associated *Flavobacterium* spp. exhibited a higher abundance of tRNAs and rRNAs, it is difficult to assert that they possess superior colonization and/or stress adaptation capabilities in comparison to other groups. Nonetheless, marine taxa may have a higher capacity to exploit nutrients in resource-limited or low-temperature environments by a *K*-strategy (66, 69).

Moreover, we evaluated genes related to resistance to metals or biocides in all genomes. A total of 84 genomes were identified, comprising 56 terrestrial, 18 host-associated, and 10 marine genomes, all of which contained genes associated with resistance to metals and biocides (Table S5). The majority (62.59%, *n* = 87) of the 139 genes were identified as resistance genes for benzylkonium (benzalkonium) chloride (BAC, biocide) and mercury (metal). In addition, two genes accounted for more resistance genes in the terrestrial (68.48%) and marine (68.75%) groups than in the host-associated group (41.94%). Even though residual BAC levels are regulated in some countries, the potential environmental crisis is expected to increase. BAC is widely used as a disinfectant in industrial, healthcare, or animal culture industries (70). The phylum Bacteroidota (formerly Bacteroidetes) is a well-characterized mercury-resistant taxon, particularly in marine environments (71, 72). Our results revealed that genes for mercury resistance are prevalent in the genomes of *Flavobacterium* spp. in terrestrial environments. These results suggest that *Flavobacterium* spp. could easily acquire BAC resistance and that this resistance ability could spread throughout the natural microbial community (73).

#### Prophages

**Fig 2:**
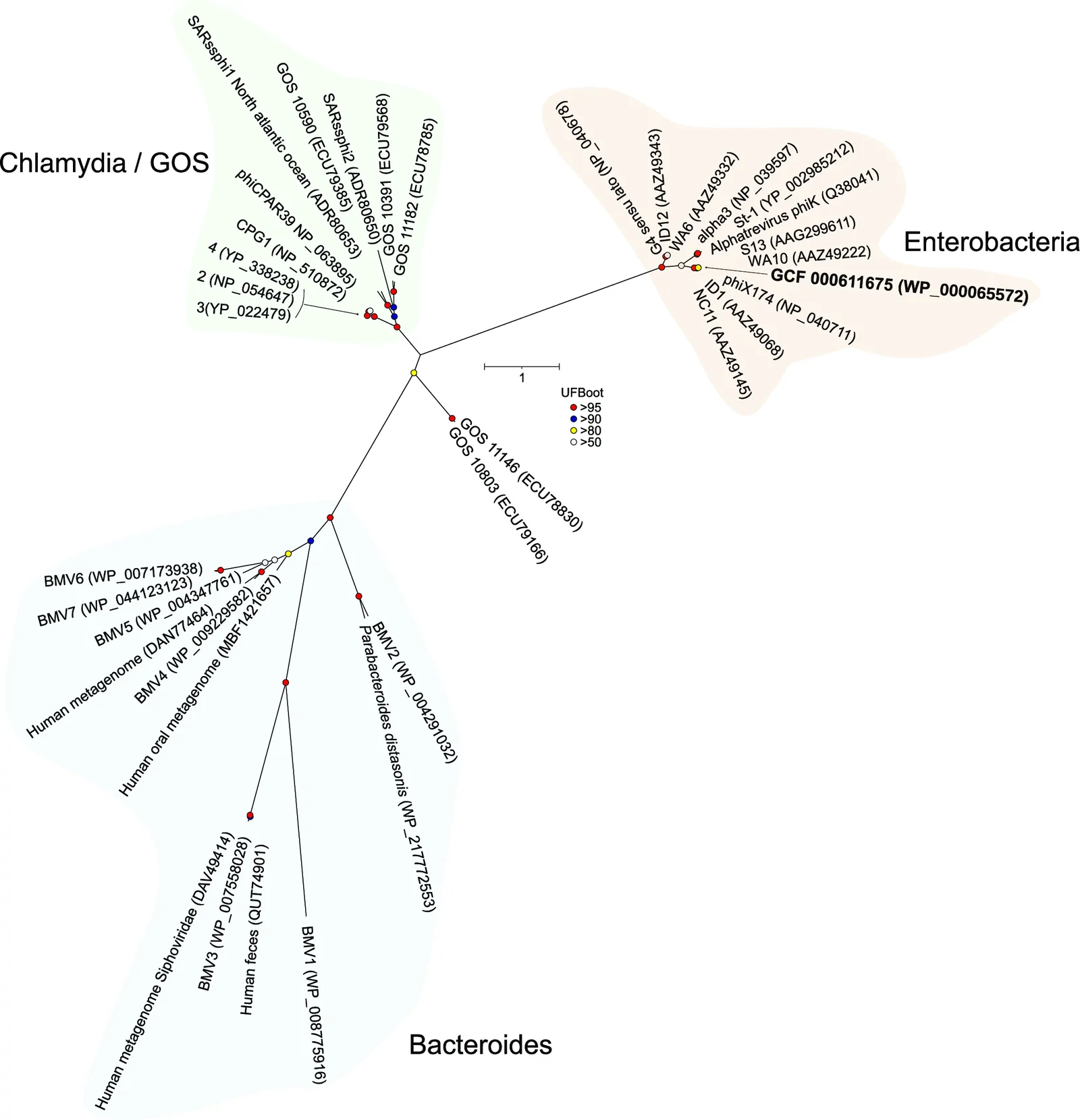
Unrooted maximum likelihood phylogenetic tree based on capsid sequences (*Microviridae*) from the *Flavobacterium* genome (GCF_000611675) identified in this study and from genomes of *Microviridae* isolated from Chlamydia, Bacteroides, Enterobacteria, and the Global Ocean Sequencing project. The phylogenetic tree was generated with ultrafast bootstrapping and the best-fit model (Q.pfam + F + R9) of amino acid substitutions. Bootstrap values on nodes are indicated by colored circles (see legend). Scale bar represents one substitution per site.

Bacteriophages, which are abundant viruses, have critical ecological functions, including roles in the control of microbial abundance and community structure as well as effects on genome plasticity in prokaryotes (74 – 76). We investigated prophage-mediated genome flexibility in *Flavobacterium*. There are only two studies (77, 78) of double-stranded DNA phages isolated from the genus, and these belong to the newly proposed family Herelleviridae (79). We detected incomplete phages in the *Flavobacterium* genomes, except for *Flavobacterium succinicans* LMG 10402 genome (GCF_000611675) isolated from an eroded salmon fin (80). *F. succinicans* LMB 10402 was classified into the host-associated group. The prophage (5,534 bp) was identified as a complete lysogenic phage using Vibrant and was closely related to the *Escherichia* phage phiX174 (NC_001422), as determined using ViPTree and VIRIDIC (see additional data at https://doi.org/10.6084/m9.figshare.22109381.v1). These results were supported by a phylogenetic analysis based on major capsid protein sequences (Fig. 2). phiX174 is a type phage of the genus *Microvirus*. In fact, Microviridae now includes two distinct subgroups, the subfamilies Bullavirinae (formerly *Microvirus*) and Gokushovirinae, with circular single-stranded DNA genomes (79, 81). Members of the two distinct groups infect enterobacteria (e.g., *Enterobacter*) and obligate intracellular parasitic bacteria (e.g., *Chlamydia*), respectively. To our knowledge, no members of the genus *Microvirus* have been identified in *Flavobacterium* spp. Krupovic and Forterre (82) reported *Microvirus*-related prophages in genomes of six species belonging to the phylum Bacteroidetes as part of the human microbiome; however, these six strains were not related to the genus *Flavobacterium*. Metagenomic studies have expanded our understanding of the diversity and essential roles of the family Microviridae in various ecosystems; however, some genera in the family have been primarily characterized from human fecal metagenome data sets and are not closely related to the genus *Flavobacterium* (83 – 85). It is possible that the members of the genus *Flavobacterium* have few lytic phages and little opportunity for the horizontal acquisition of new genes via phages. Furthermore, it is unclear how and why Microviridae phages were incorporated into the genome (GCF_000611675) of *F. succinicans* LMG 10402 in salmon.

#### Proteorhodopsin

**Fig 3:**
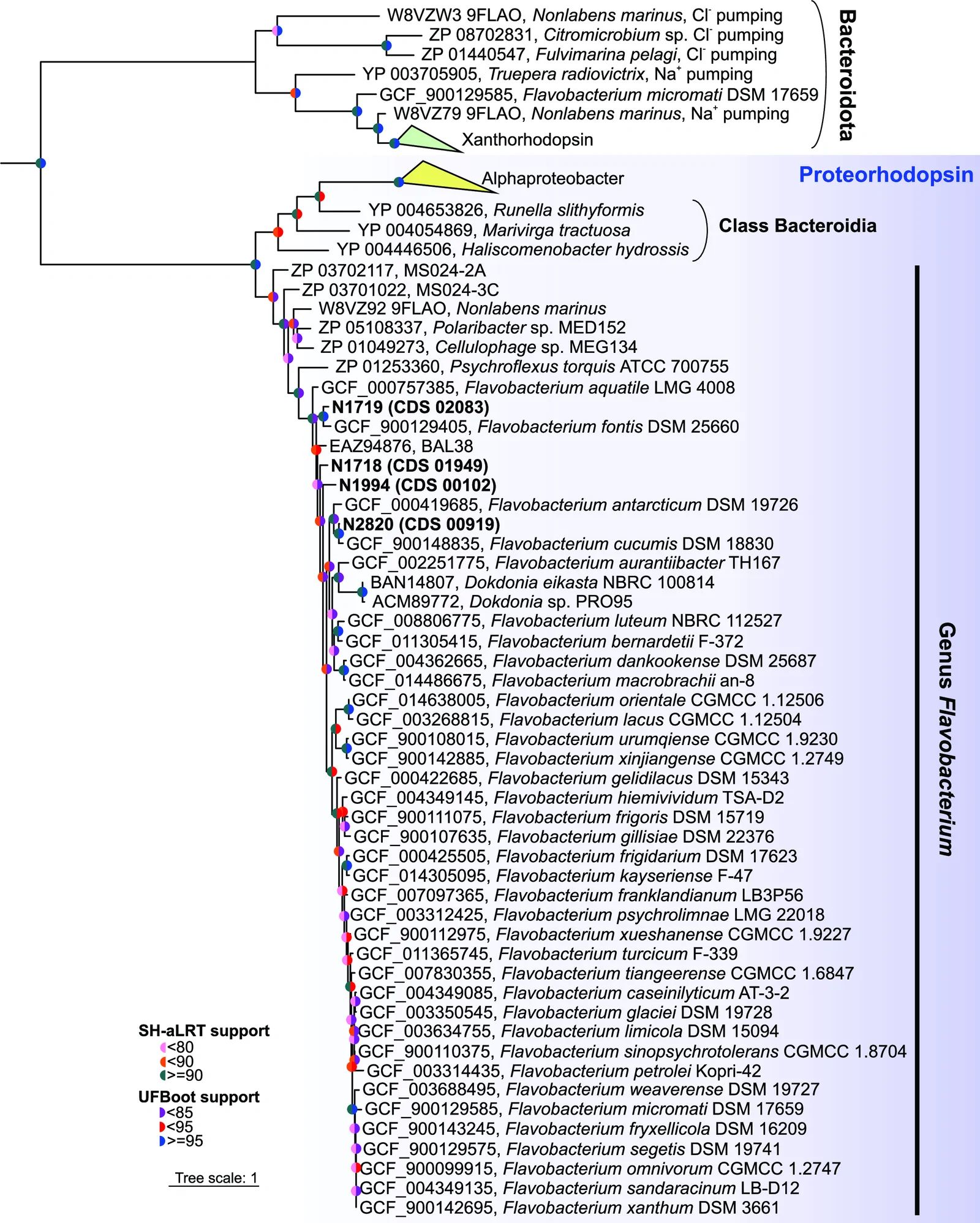
Maximum likelihood phylogenetic tree of proteorhodopsin sequences from the *Flavobacterium* genomes analyzed in this study and reference sequences, including xanthorhodopsin, chloride-pumping, and sodium pumping rhodopsin genes, in *Bacteroidota*. Support was evaluated by SH-aLRT and Ultraboost, and the tree was generated with the best-fit model (Q.pfam +F + R3) of amino acid substitutions. Bootstrap support values are shown as colored half-circles at nodes (see the legend). Scale bar represents one substitution per site.

As photoactive membrane-embedded opsins, rhodopsin displays extensive versatile functions in aquatic taxa, especially in oceans (86). Environmental metagenomics has revealed that various types of rhodopsin are broadly distributed in all domains, including viruses (87), with recent reports of its evolution and function in bacteria and Archaea (88, 89). There is experimental evidence for a proteorhodopsin (PR)-containing strain in the genus *Flavobacterium* isolated from coastal waters (90). In fact, phototrophic growth has been examined in three marine bacterial strains belonging to the class Flavobacteria isolated from surface seawater (91). Other genera (e.g., *Dokdonia*, *Polaribacter*, and *Nonlabens*) in the class Flavobacteria contain functionally diverse rhodopsins. In fact, in nutrient-poor marine environments, marine *Flavobacterium* with PR genes may have an ecological benefit with respect to light-stimulating growth or osmotic stress elimination (92, 93). Nevertheless, investigations of photoheterotrophic traits in the genus *Flavobacterium* are limited even though the class Flavobacteria is involved in organic matter cycling in the ocean (2). Therefore, we screened the genomes of strains isolated in this study. Four strains (N1718, N1719, N1994, and N2820) in the terrestrial group harbored a green-light absorbing PR gene in the genome (Fig. 1 and 3). PR genes from four strains encoded 231–244 amino acids, sharing a sequence similarity of 82.63%. Four genes clearly belonged to the genus *Flavobacterium*, except for one in the genus *Dokdonia*. PR genes of strains N1719 and N2820 were closely related to a previously isolated strain (Fig. 3). In addition, based on sequence similarity and phylogenetic relationships, four PRs were affiliated with a DTE-motif rhodopsin (Fig. 3). Additionally, the gene for betacarotene 15,15′-dioxygenase (*blh*) was detected in the genomes and was adjacent to the PR gene. However, the genomes of four strains lacked genes (*crtEIY*) involved in retinal biosynthesis, except for *crtB* (phytoene synthase). Of note, only 16 strains isolated in this study had a gene encoding 4,4′-diapophytoene desaturase (CrtN). CrtN is a component of the C_30_ carotenoid (e.g., staphyloxanthin and yellow pigment) biosynthesis pathway (*crtMNOPQ*) (94).

**Fig 4:**
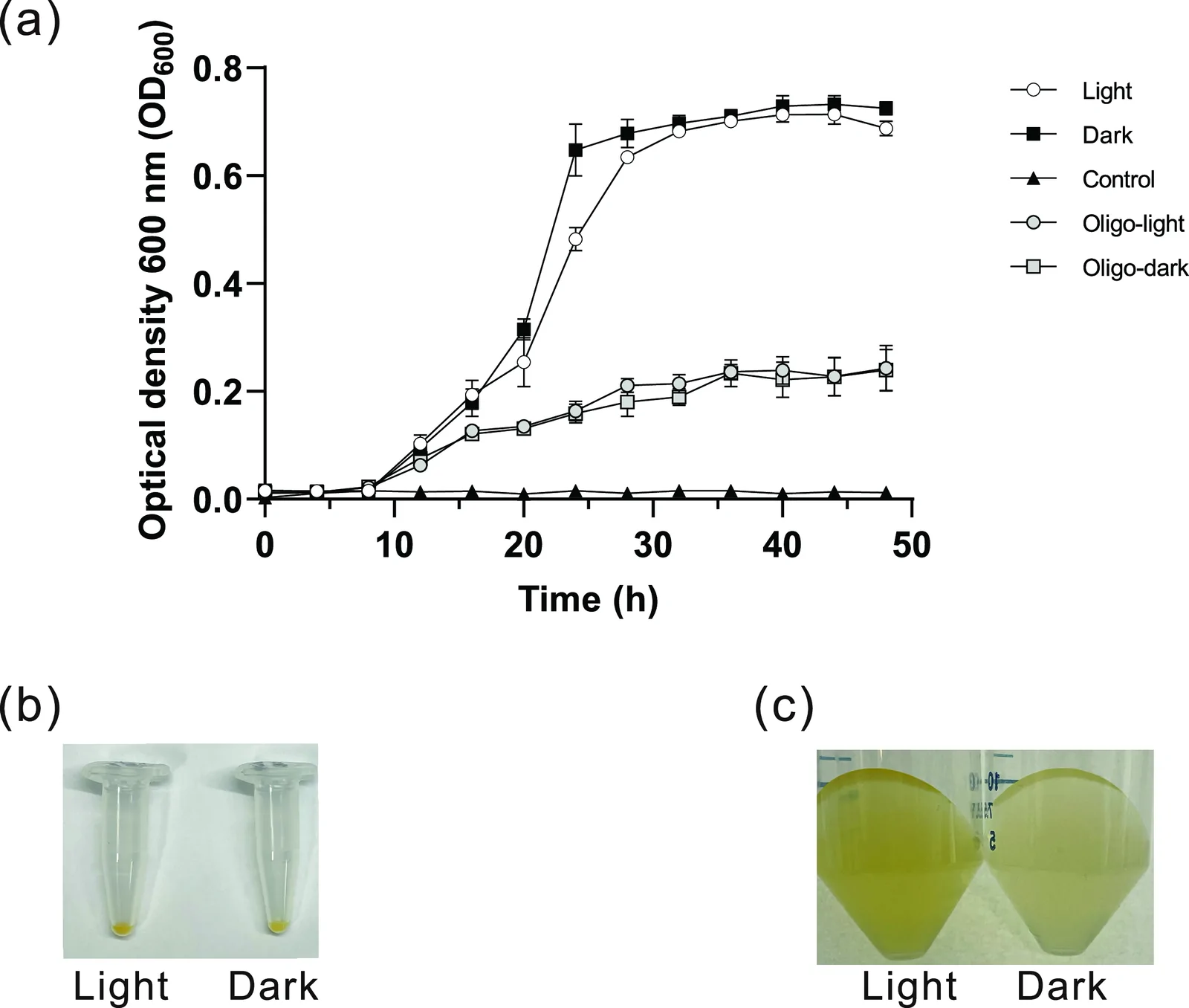
Growth of *Flavobacterium* sp. N2820 cultured in light and dark conditions. Cells were grown in R2A broth at 26°C with agitation (180 rpm). Cell density was determined by optical density 600 nm (OD_600_) (**a**). Error bars denote the standard deviation from triplicate cultures. The color of centrifuged strain N2820 cell pellets (**b**) and liquid medium (**c**) after culture under light and dark conditions (**b**). Control and oligo denote were no cell and nutrient limitation condition, respectively. See Materials and Methods.

To evaluate the effect of light on the growth of the four strains, we compared the growth in light and dark culture conditions on R2A agar plates. Growth inhibition of strain N1718 was observed under light conditions. No difference in growth was detected between light and dark conditions for strains N1994 and N1719. Light-stimulated growth was only observed in strain N2820. To verify this observation, the strain N2820 was cultivated in R2A broth and nutrient-limited media under light and dark conditions with agitation. However, no recognizable difference in cell growth was observed under different culture conditions (Fig. 4). Interestingly, we found that strain N2820 cells under light conditions were darker than cells under dark conditions (Fig. 4) with agitation. Taken together, these results suggest that betacarotene production will be improved under light or oxygen (i.e., aeration) stress (95). In addition, it is not apparent that the aforementioned strains have a photoheterotrophic lifestyle in which they obtain energy from light. Neverthelss, light can stimulate the growth of zeaxanthin-producing *Flavobacterium* spp. under anoxic and oligotrophic conditions (96). By contrast, our genome analysis did not reveal evidence for biosynthesis capabilities for retinal and other carotenoids (except betacarotene). Interestingly, zeaxanthin-producing strains have been isolated from extreme cold environments (i.e., glaciers). Although this hypothesis is supported by only a single study, pigment compositions, including various types of carotenoids, might be related to temperature sensitivity in the genus *Flavobacterium*. In addition, there may be unknown biosynthetic pathways and enzymes in *Flavobacterium* spp. related to carotenoid production (94, 97, 98).

### Genome diversity and evolution

As mentioned above, *Flavobacterium* genomes might have genes related to adaptability to heterogeneous environments. In addition, the genetic plasticity of micro-organisms via HGT or recombination events is closely related to ecological diversity, adaptation, and evolution (19, 21). Therefore, we evaluated genetic flexibility in the genus *Flavobacterium* by detecting HGT and recombination events. Genomes of other genera (*n* = 12, e.g., *Galbibacter*, *Joostella*, *Marixanthomonas*, *Pustulibacterium*, *Sinomicrobium*, and *Imtechella*) were included in the HGT analysis. Only 279 HGT events were detected in the genus and species (Table S6 and Fig. S9). The values for amino acid identity ranged from 74.46% to 100% (avg. 84.08%).

We estimated recombination events in *Flavobacterium* genomes. We obtained 1–68 predicted recombination events per genome, except for two genomes [*Flavobacterium plurextorum* CCUG 60112 (GCF_002217395) isolated from farmed rainbow trout (*Oncorhynchus mykiss*) and strain N2469 isolated from the gut of Korean rockfish (*Sebastes schlegelii*)]. The average number of recombination events in host-associated genomes (28.53) was slightly higher than those in terrestrial (23.65) and marine (23.00) genomes. However, there were no significant differences in combination events with respect to habitat type (Kruskal–Wallis test, *P* = 0.4615). Three key model parameters (posterior mean values) were estimated: *R*/*θ* = 1.016 (0.996–1.042), *δ* = 51.89 bp (51.12–52.59 bp), and *ν* = 0.065 (0.064–0.065) (i.e., the ratio of recombination and mutation rates, the length of recombinant fragments, and the probability that each site of a recombinant fragment is mutated, respectively). In addition, the mutation rate was approximately 0.985 (*θ*/*R*). Each recombination event involved approximately 3.361 substitutions. The ratio of recombination to mutation was 3.413 (*r*/*m* = *R*/*θ* × *δ* × *ν*) (99). This suggests that recombination events generate about 3.4 times more substitutions than point mutations. The mutation rate was lower in marine *Flavobacterium* genomes (0.821) than in other groups (0.909 and 0.979 for terrestrial and host-associated genomes, respectively). However, the ratios of recombination to mutations for host-associated genomes (4.995) were higher than those of other groups (4.651 and 3.658 for marine and terrestrial genomes, respectively) and all *Flavobacterium* genomes. This indicates that homologous recombination might contribute more substantially to host-associated *Flavobacterium* genomes than to genomes in other groups (100, 101). Furthermore, this observation is supported by the numerical differences in transposable elements (e.g., transposases) among groups (host-associated: 22.24; terrestrial: 11.55; marine: 14.33, calculated by the total number of transposases per number of genomes in each group) (102). Taken together, these results indicate that recombination events might play a more critical role than mutation and lateral gene transfer in the evolution of the genus *Flavobacterium* (103).

### Concluding remarks

We performed a pangenome analysis of 187 genomes of *Flavobacterium* spp., including whole-genome sequences of 20 newly isolated strains from various sources and publicly available genome sequences. Although genes for metabolic pathways did not differ with respect to isolation sources, we found that variation in genomic traits could be important in understanding diversity, evolution, and adaptation in the genus *Flavobacterium*. In addition, we deduced that recombination events contribute to genomic diversity in *Flavobacterium* spp., without substantial genome expansion. We found significant variation in the tRNA ratio as well as a significant positive correlation between the genome size and noncoding density. In addition, we only detected PR genes in terrestrial *Flavobacterium*. Analyses of PR in marine *Flavobacterium* are limited by the small number of isolated strains. There may be a great number of unidentified members of the genus *Flavobacterium* in marine environments. GH families in *Flavobacterium* genomes have diverse catalytic activities and/or novel functional mechanisms for biotechnological applications; these families are potential targets for the development of antiinfluenza drugs, such as Tamiflu. Further insights into the evolution and adaptation against a broad range of environmental conditions would be gained through more detailed physiochemical characterization along with analyses of genomic traits.

## MATERIALS AND METHODS

### Isolation and genome sequencing

To isolate bacterial strains, environmental samples were collected from tidal flats, freshwater, wastewater treatment plants, soil, and the guts of fish and shellfish in South Korea. Each sample was separately diluted, spread onto Reasoner’s 2A Agar (R2A; Difco, Franklin Lakes, NJ, USA) or Marine Agar 2216 (Difco), and incubated at 30°C for 1 week under dark conditions. Single colonies were isolated through several subcultures (at least five) via transfer to new R2A or MA plates. To select bacterial strains belonging to the genus *Flavobacterium*, the 16S rRNA gene of each isolate was sequenced and evaluated by phylogenetic analyses, as described in our previous studies (104, 105). Finally, 20 strains belonging to the genus *Flavobacterium* were successfully isolated (see Table S6).

For complete genome sequencing, the genomic DNA of each strain was extracted using a Monarch Genomic DNA Purification Kit (New England Biolabs, Frankfurt, Germany) according to the manufacturer’s instructions. Then, a gDNA library was prepared using the SMRTbell (Single-Molecule Real-Time sequencing) Kit (Pacific Biosciences, Menlo Park, CA, USA) following the protocol for the PacBio RSII platform at DNA Link (Seoul, South Korea) and Theragen Bio (Seongnam, South Korea). All sequence reads from each strain were completely assembled using Flye (v2.8.3) (106) or HGAP3 (107) with default parameters. Plasmid sequences were found in only two strains (N1861 and N2155). Quality parameters (e.g., completeness and contamination) for all assembled genomes were estimated using CheckM (v1.2.0) (27).

### Genome annotation and functional prediction

For the (pan)genome analysis, publicly available complete or draft genomes were downloaded from Genome Taxonomy Database (GTDB, accessed on July 2022) (Table S2). One hundred sixty-seven genomes were obtained by setting “GTDB species representative only” and “NCBI type material only.” Genes for all genomes (*n* = 187 including 20 strains isolated in this study) were predicted and annotated using prokka (v 1.14.5) with default parameters. For KEGG Orthology (KO numbers) and COGs database analyses, BlastKOALA (108) and RPS-BAST (reverse-position-specific BLAST) (109) were used. To infer putative metabolic pathways and cellular functions, KEGG Mapper (KEGG mapping tools) was applied to the *Flavobacterium* genomes (110). Ribosomal RNAs and transfer RNAs (tRNAs) were isolated using barrnap and tRNAscan-SE, as described previously (105). To analyze carbohydrate utilization genes, predicted protein-coding sequences (CDSs) were evaluated using the dbCAN2 annotation tool (111). Prophage sequences of all genomes were identified using VIBRANT (112). For additional annotation, classification, and phylogenetic analyses of complete phages, we used ViPTree (113), VIRIDIC (114), and VICTOR (115) for protein-based hierarchical clustering. Antibacterial biocide and metal resistance genes were identified using the BacMet database (116).

### Pangenome and phylogenomic analyses

For a pangenome analysis using Roary (v3.11.2), the GFF3 file of all genomes was downloaded from GenBank (NCBI) or produced by Prokka (see above) with default parameters (117). The Roary results were visualized using the Pagoo package (118) in R (https://www.r-project.org/). Phylogenomic analysis of the genomes, including those for closely related genera (*Galbibacter* and *Joostella*), was performed based on single-copy marker genes as described previously (105, 119). Briefly, genomes containing at least 20% of markers (38 of 161 markers) based on the concatenated CDSs were included. Individual homologous CDSs were aligned using PASTA (v.1.8.3) with default parameters (120), and a maximum likelihood tree was constructed using IQ-TREE2 (121) with the best-fitting substitution model (GTR + F + I + I + R8) determined using ModelFinder (122). The reliability of each internal branch was evaluated by the SH-like approximate likelihood ratio (SH-aLRT, -alrt 1000) (123) and ultrafast bootstrap (UFBoot, -bb 1000) (124). ClonalFrameML and MetaCHIP were applied to infer recombination events simulated with within-population and HGT events, respectively (99, 125). Average nucleotide identity and average amino acid identity for all *Flavobacterium* genomes were calculated as described previously (105).

### Phylogenetic analyses of proteorhodopsin and capsid genes

Proteorhodopsin-like gene sequences were identified in all *Flavobacterium* genomes, including genomes of new isolates in this study. Related bacteriorhodopsin sequences were obtained from publicly available data (119). Capsid sequences of *Microviridae* were obtained from previous studies (82, 83). An amino acid sequence alignment was generated using PASTA with default parameters. The maximum likelihood tree was inferred using IQ-TREE2 with SH-aLRT and UFBoot (-alrt 100 -bb 1,000) (see above).

### Culture conditions for PR-containing strains

Four *Flavobacterium* strains containing PR genes were cultured at 26°C under continuous white light (~300 µmol photons m^−2^s^−1^) or in the dark (wrapped in aluminum foil) with shaking at 180 rpm. For oligotrophic cultivation (i.e., nutrient limitation), bacterial cells were grown in artificial freshwater medium supplemented with 0.5% (vol/vol) peptone and 1× vitamin mixture (105). The pH of the medium was adjusted to 7.5 with KOH and 5 mM HEPES buffer (pH 7.0). Unless specified otherwise, all strains were grown in R2A broth medium. For static culture, cells were grown on R2A (Difco) agar plates. Cell growth was measured by optical density at 600 nm (OD_600_). All culture experiments were performed in triplicate.

### Statistical analysis

Genomic trait profiles for isolates from different sources (i.e., habitats) in the genus *Flavobacterium* were compared by nonparametric analysis of variance (ANOVA and Kruskal–Wallis tests), followed by Dunnett’s multiple comparison tests using GraphPad Prism 9 (GraphPad Software, San Diego, CA, USA; www.graphpad.com). Sequentially, if a significant difference was expected, additional Welch’s *t*-tests as estimation statistics (i.e., difference between means) were performed for comparisons between two groups (126). Correlation matrix analysis (between number of CDSs and genome length) was calculated using Pearson correlation coefficients supplied by GraphPad Prism 9. Data were visualized using GraphPad Prism nine and Origin Pro 2022 (OriginLab Corporation, Northampton, MA, USA).

## Data Availability

The complete genome sequences of the strains isolated in this study were deposited in GenBank under accession numbers CP091861 and CP109992-CP110013. All isolated strains have been deposited in the National Institute of Biological Resources (curator, I.-T. Cha) (see Table S2). The whole-genome sequences have been deposited in the BioProject database under accession numbers PRJNA796153 and PRJNA891109. Additional data that support the findings of this paper are available in Figshare.

## SUPPLEMENTAL MATERIAL

Supplemental figures spectrum.01003-23-s0001.pdf Fig. S1 to S9.

Supplemental legends spectrum.01003-23-s0002.docx Legends for supplemental tables and figures.

Table S1 spectrum.01003-23-s0003.xlsx Quality of full-length sequenced genomes, as determined using CheckM, for strains isolated in this study.

Table S2 spectrum.01003-23-s0004.xlsx Detailed information on reference genomes (from GTDB) included in this study and newly isolated strains.

Table S3 spectrum.01003-23-s0005.xlsx Summary statistics for the genomes analyzed in this study.

Table S4 spectrum.01003-23-s0006.xlsx Genes involved in carbohydrate assimilation in all Flavobacterium sp. genomes included in this study.

Table S5 spectrum.01003-23-s0007.xlsx Antibacterial biocide and metal resistance genes in 187 genomes analyzed by BacMed.

Table S6 spectrum.01003-23-s0008.xlsx Horizontal gene transfer predicted by MetaCHIP.

ASM does not own the copyrights to Supplemental Material that may be linked to, or accessed through, an article. The authors have granted ASM a non-exclusive, world-wide license to publish the Supplemental Material files. Please contact the corresponding author directly for reuse.
