# Supplementary figures and images for: Pangenome analysis provides insights into the genetic diversity, metabolic versatility, and evolution of the genus Flavobacterium
Source: Microbiol Spectr. 2023 Aug 18;11(5):e01003-23. doi: 10.1128/spectrum.01003-23 (PMC10655711; doi:10.1128/spectrum.01003-23)

(a)

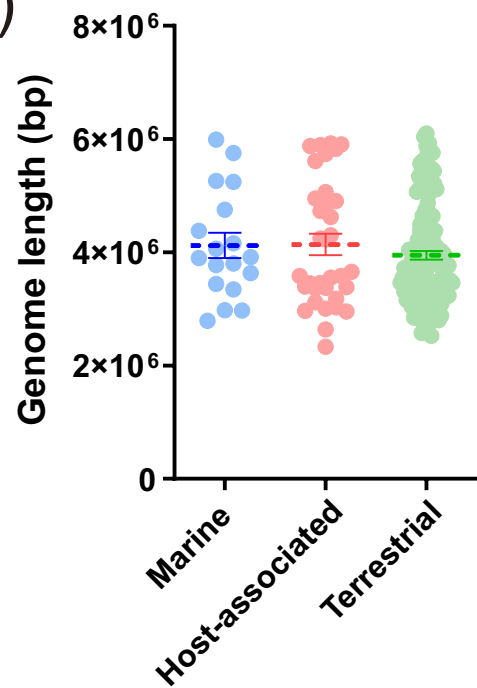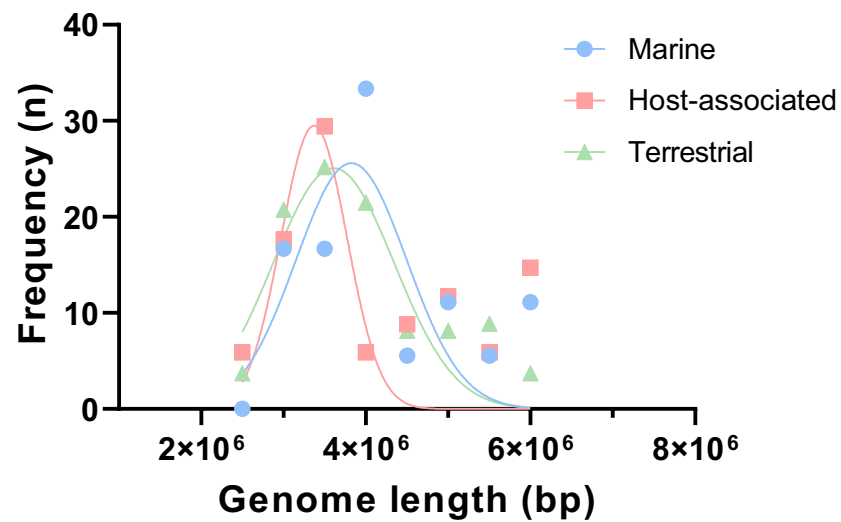

(b)

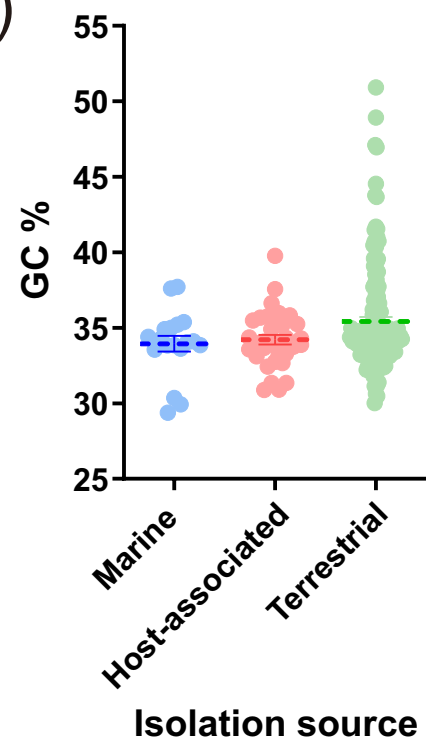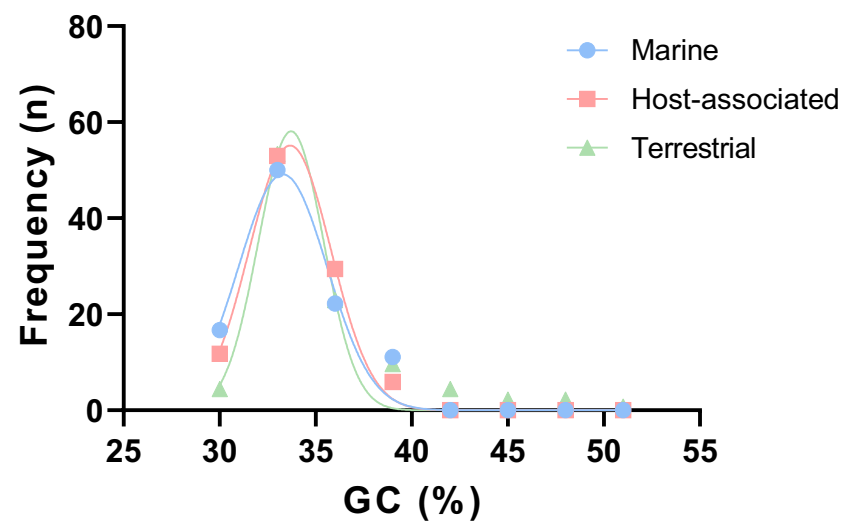

(a)

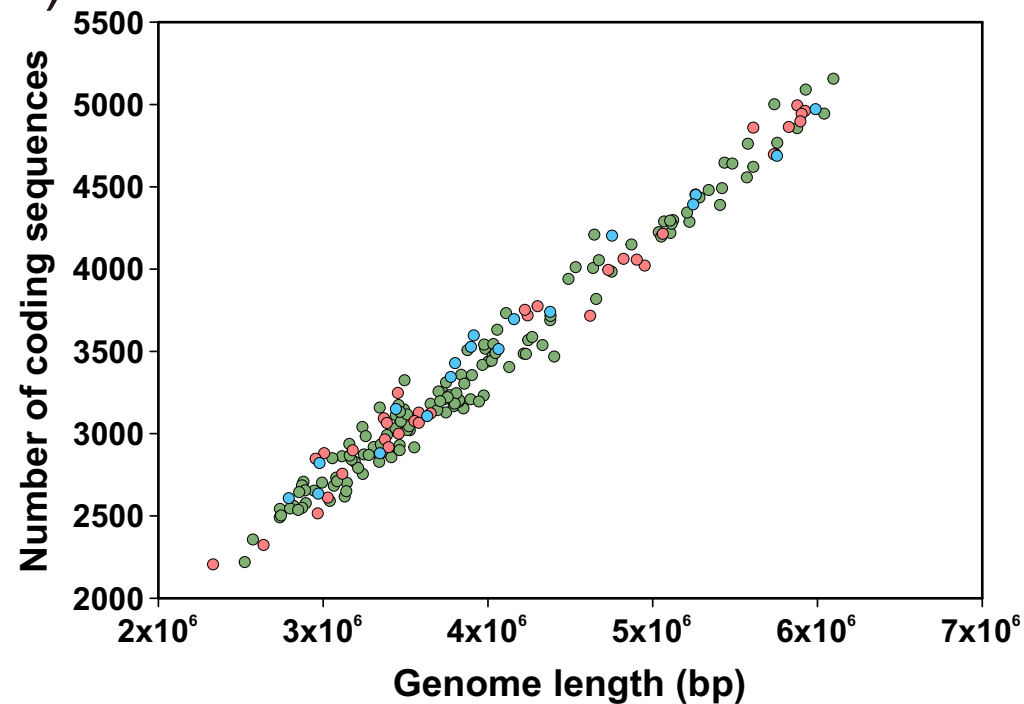

(b)

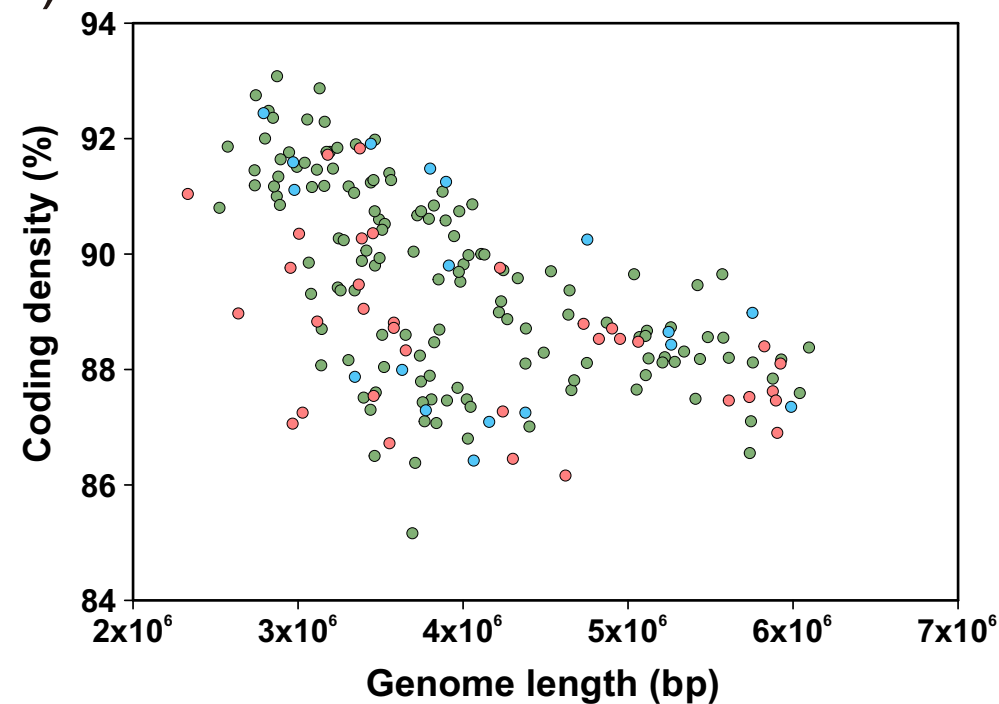

(c)

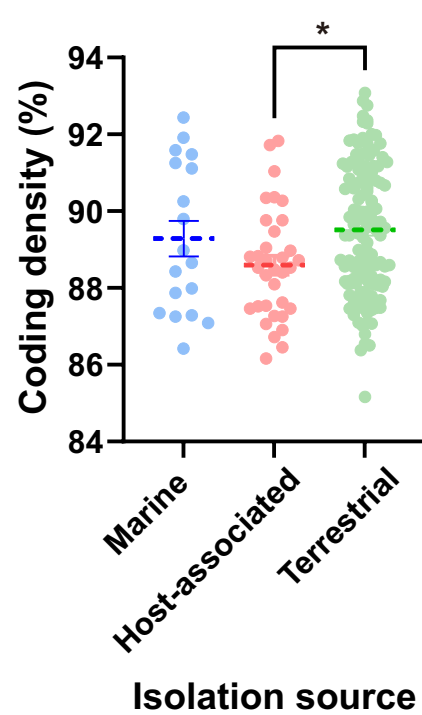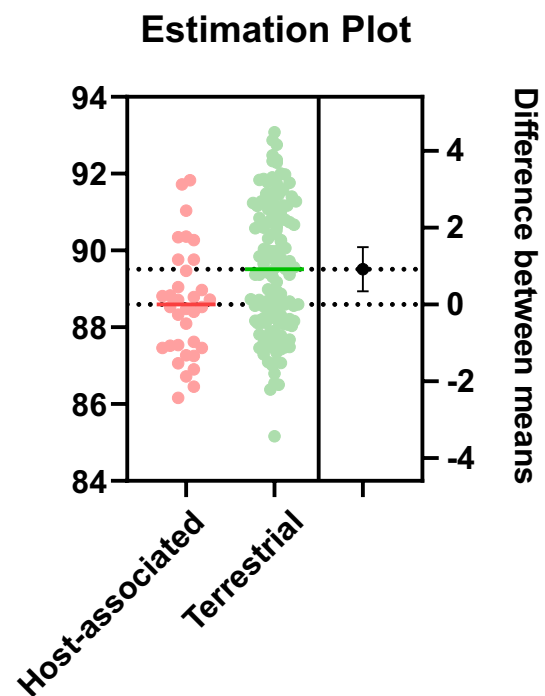

Fig. S2

**Fig. S3a**

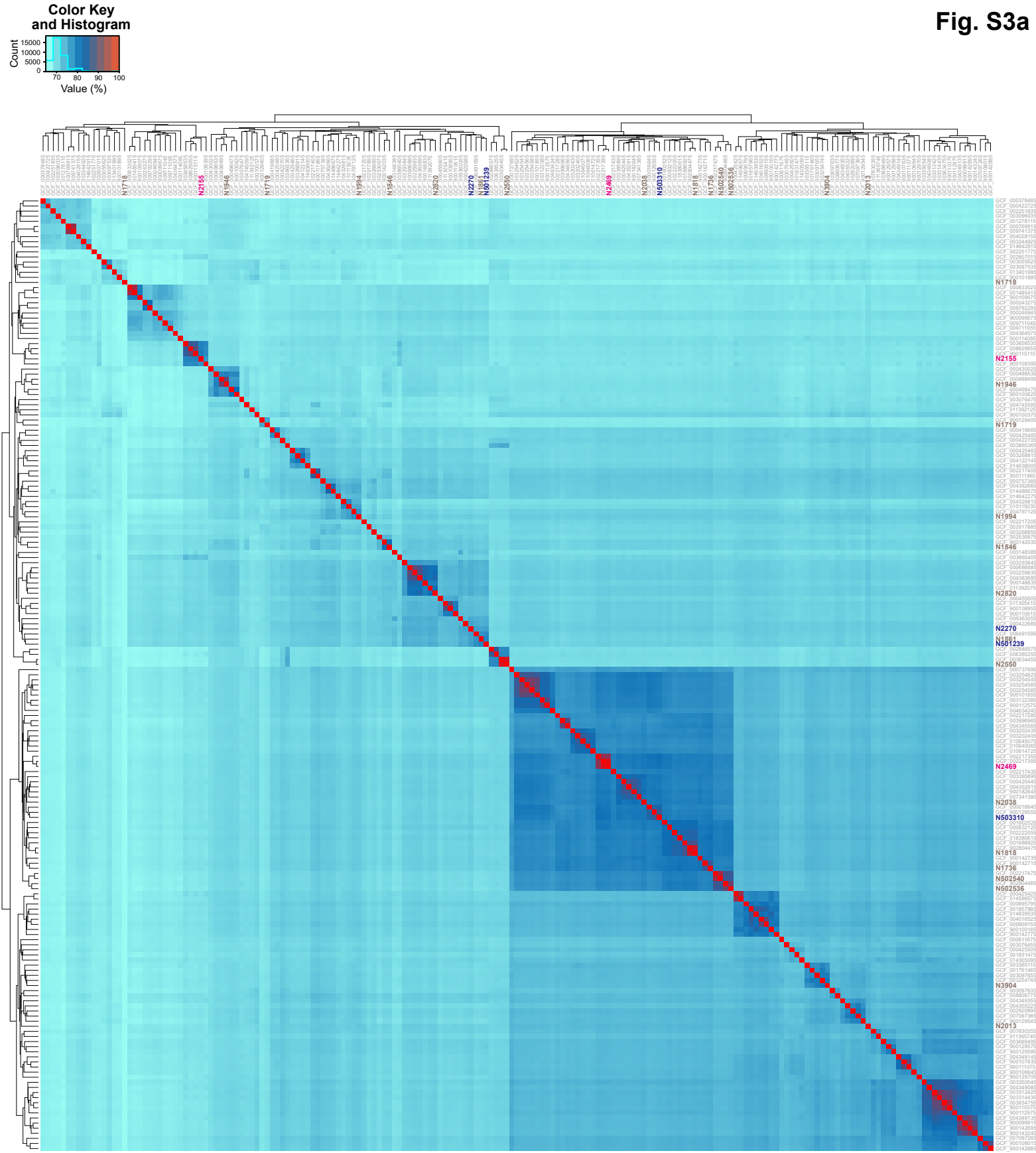

Fig. S3b

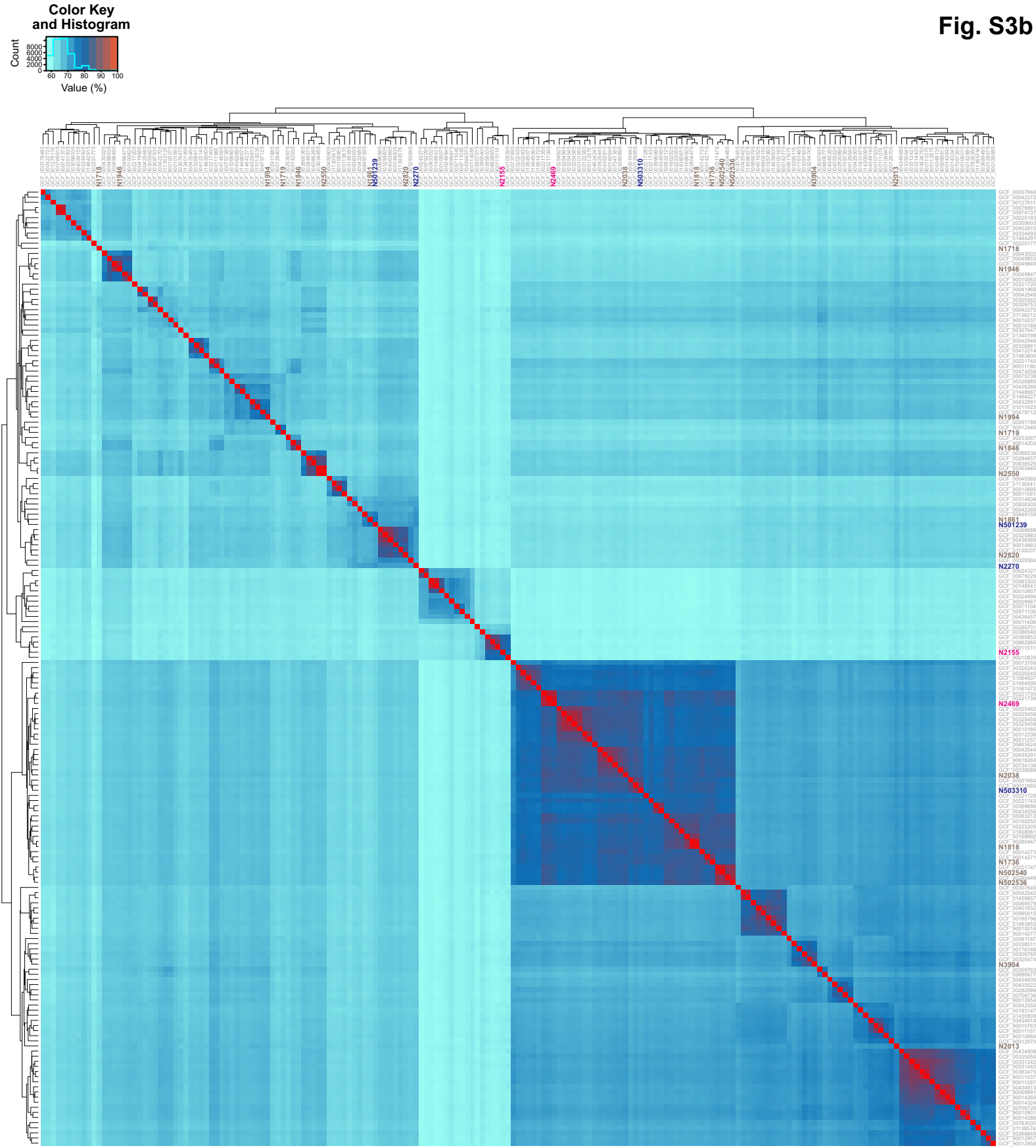

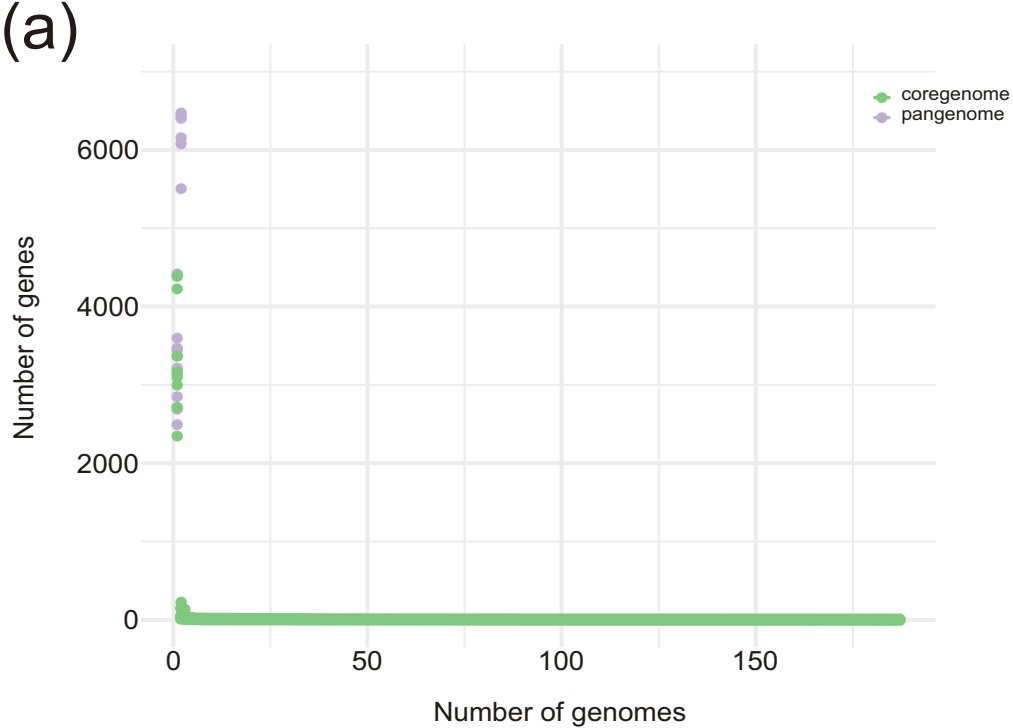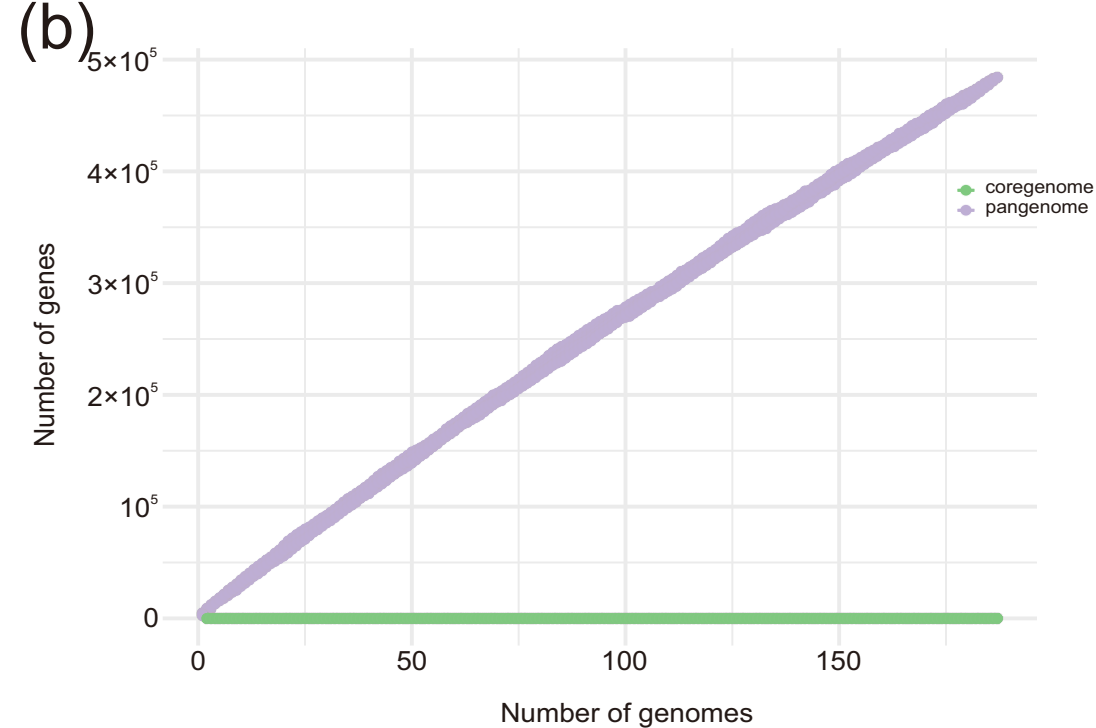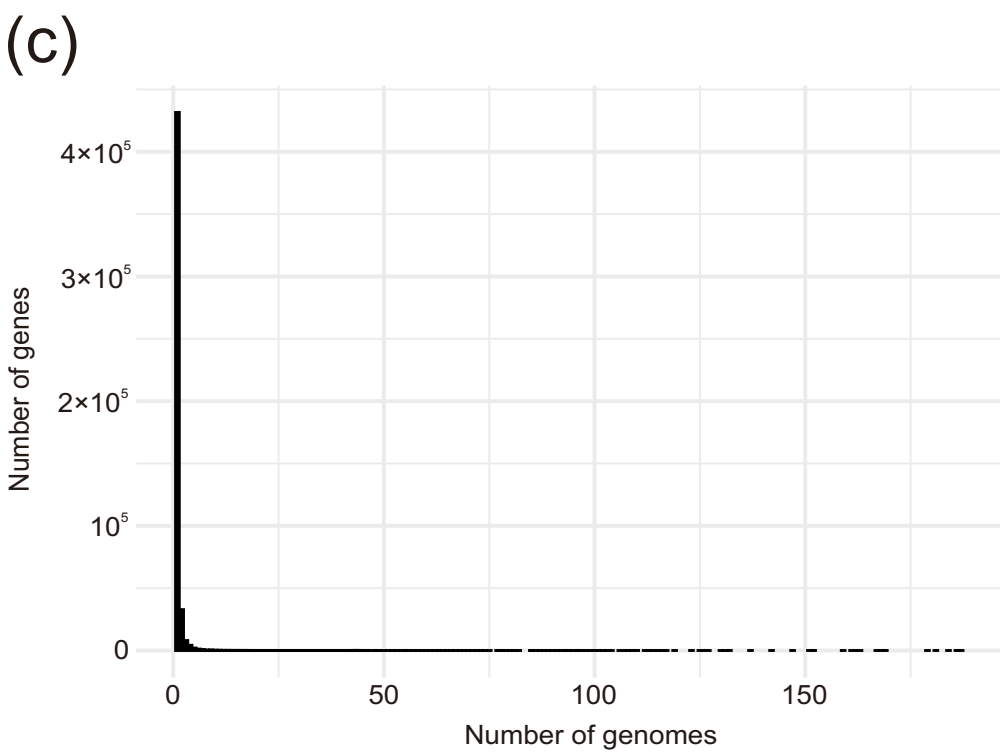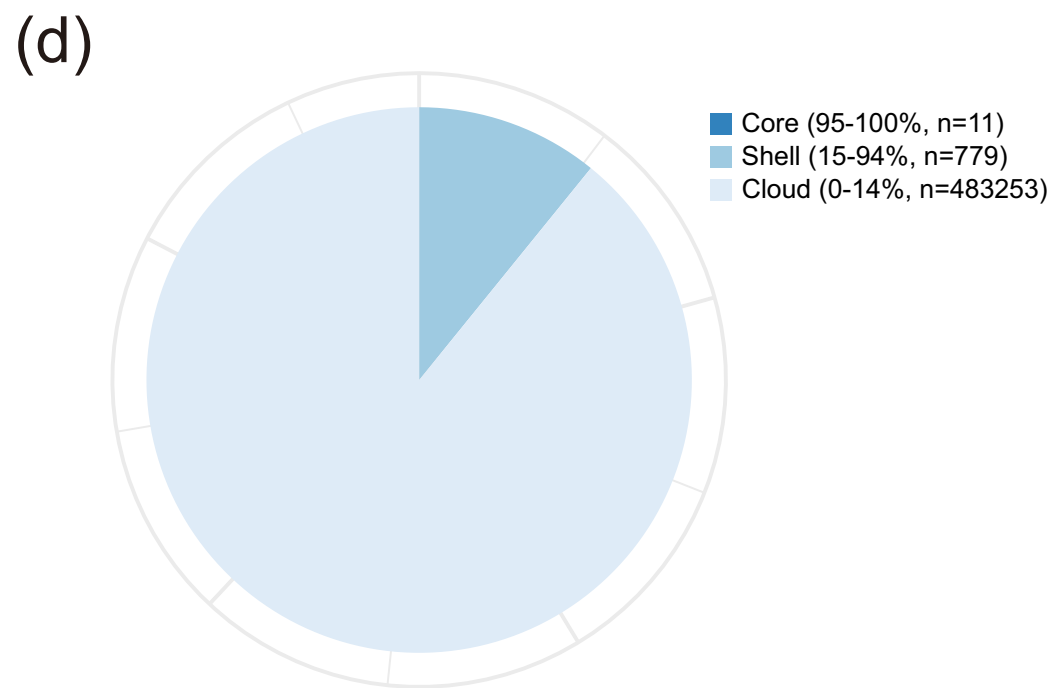

Fig. S4

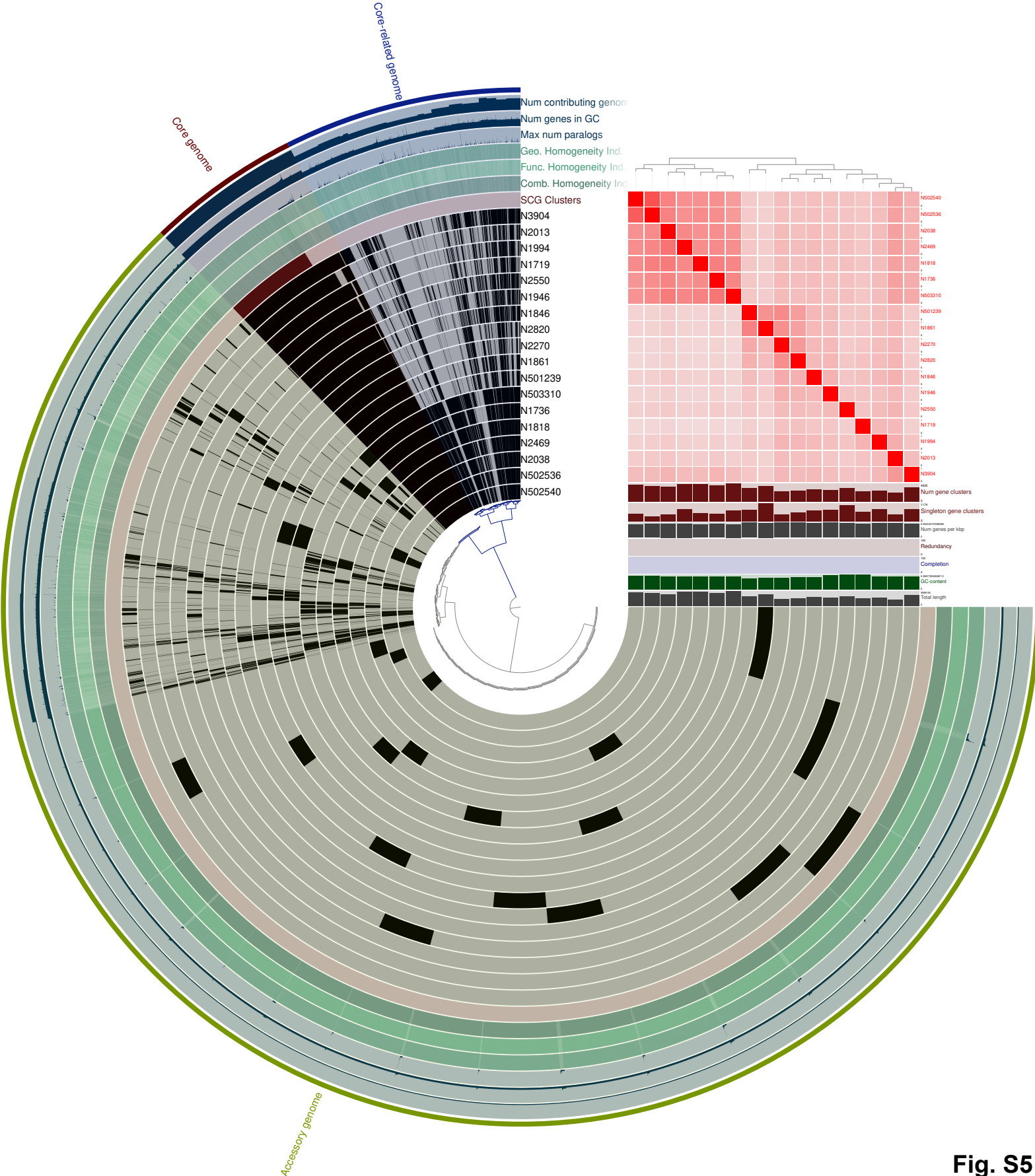

Fig. S5

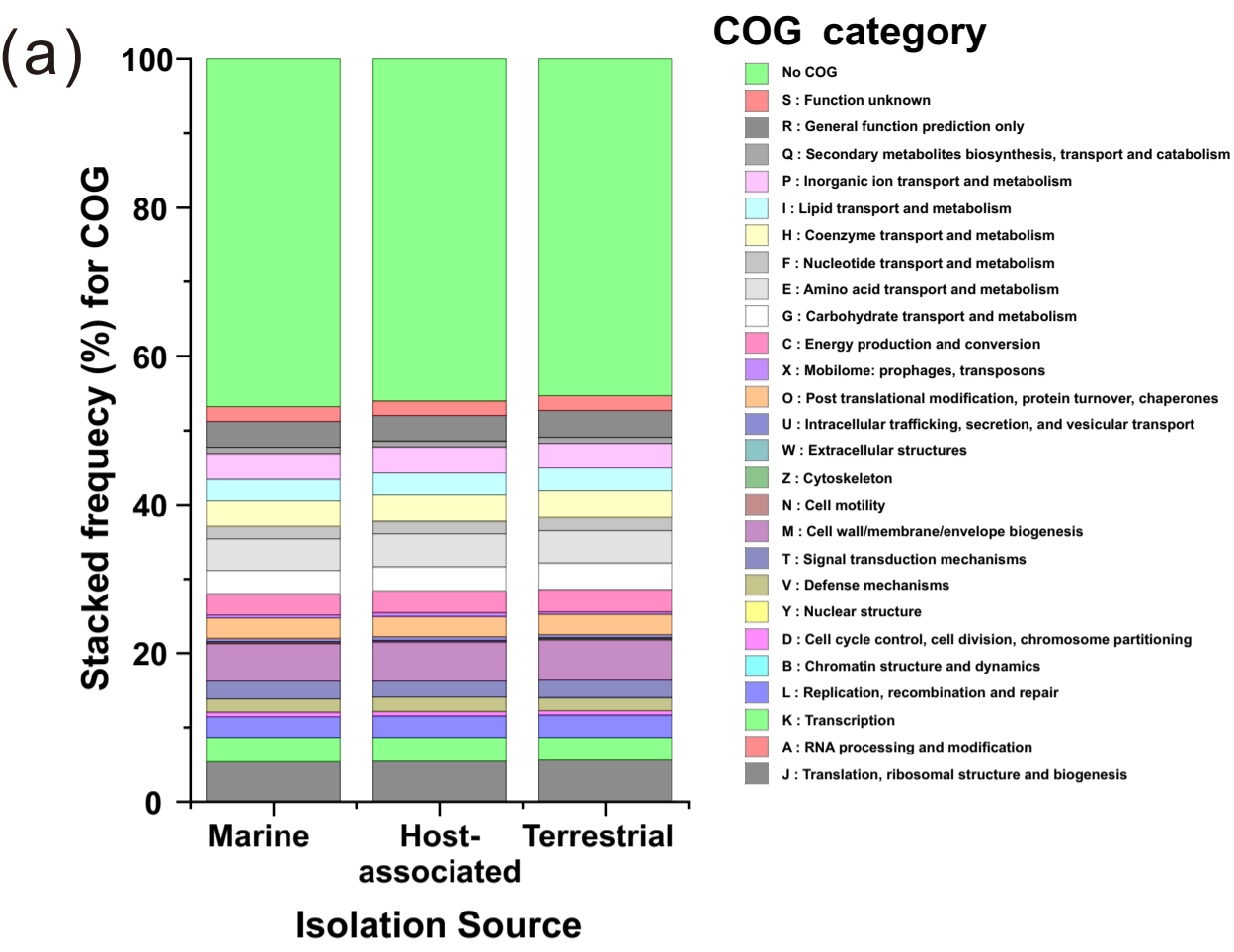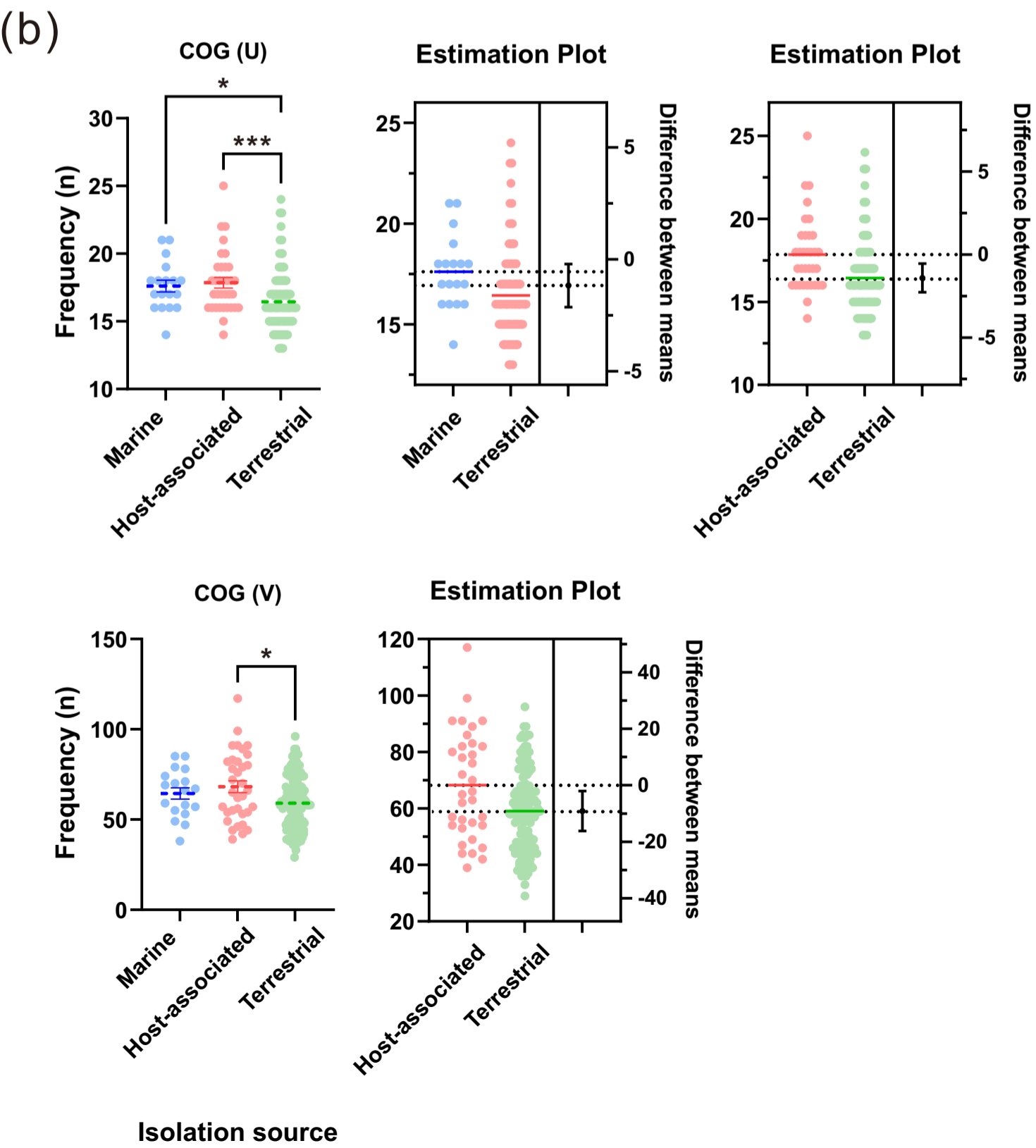

Fig. S6

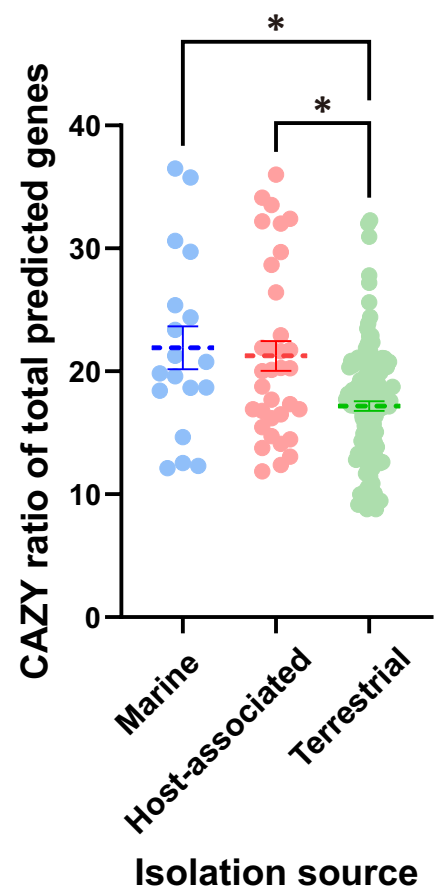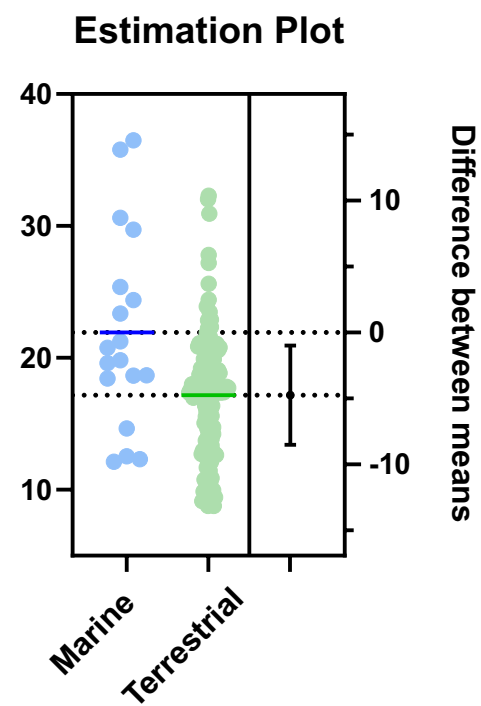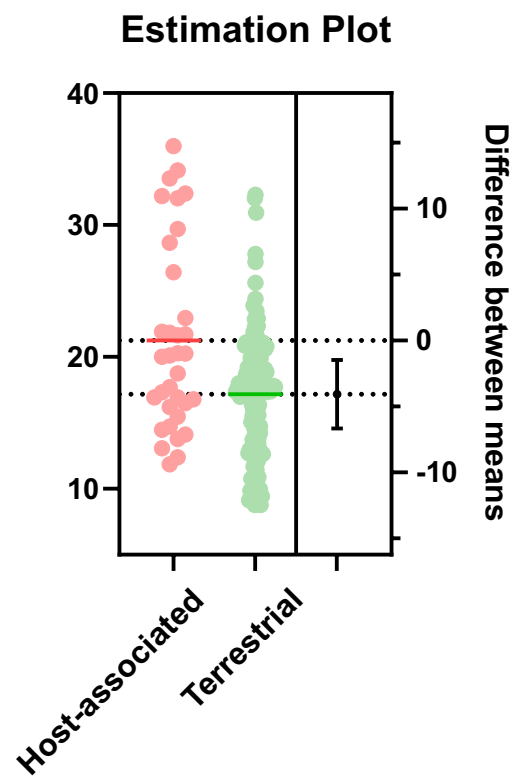

Fig. S7

(a)

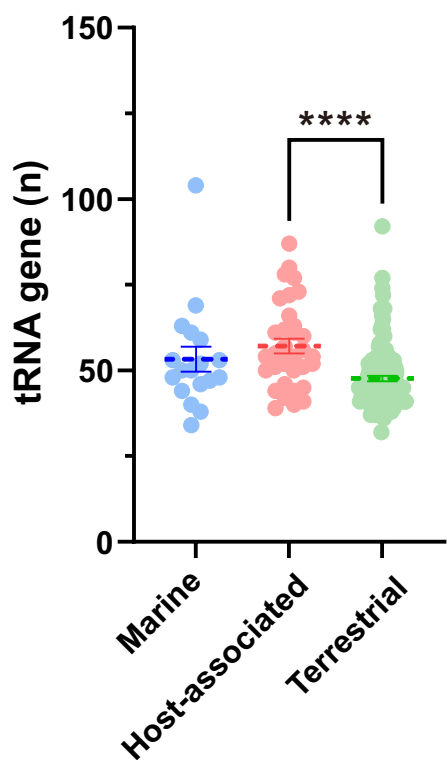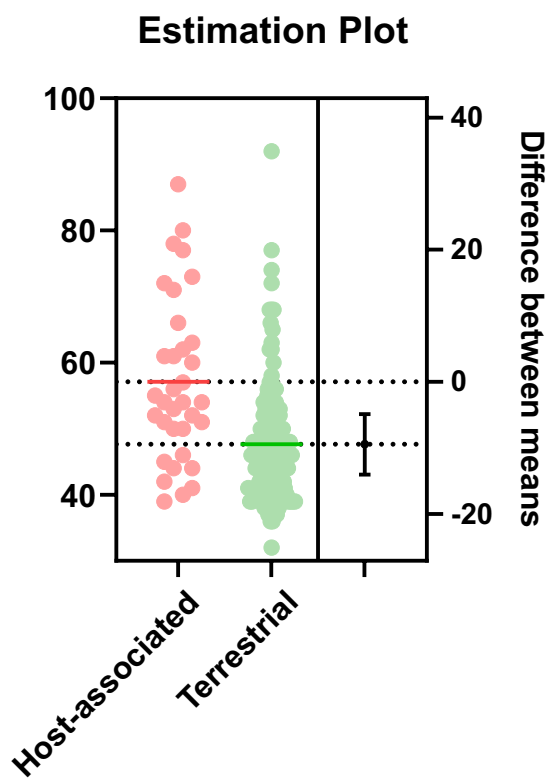

(b)

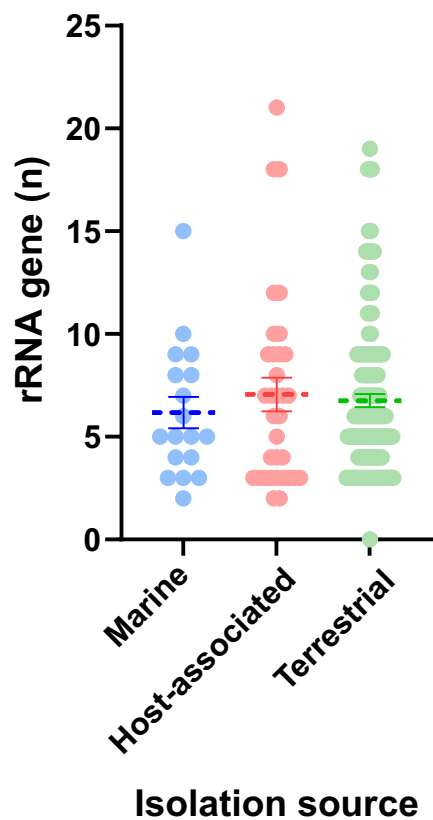

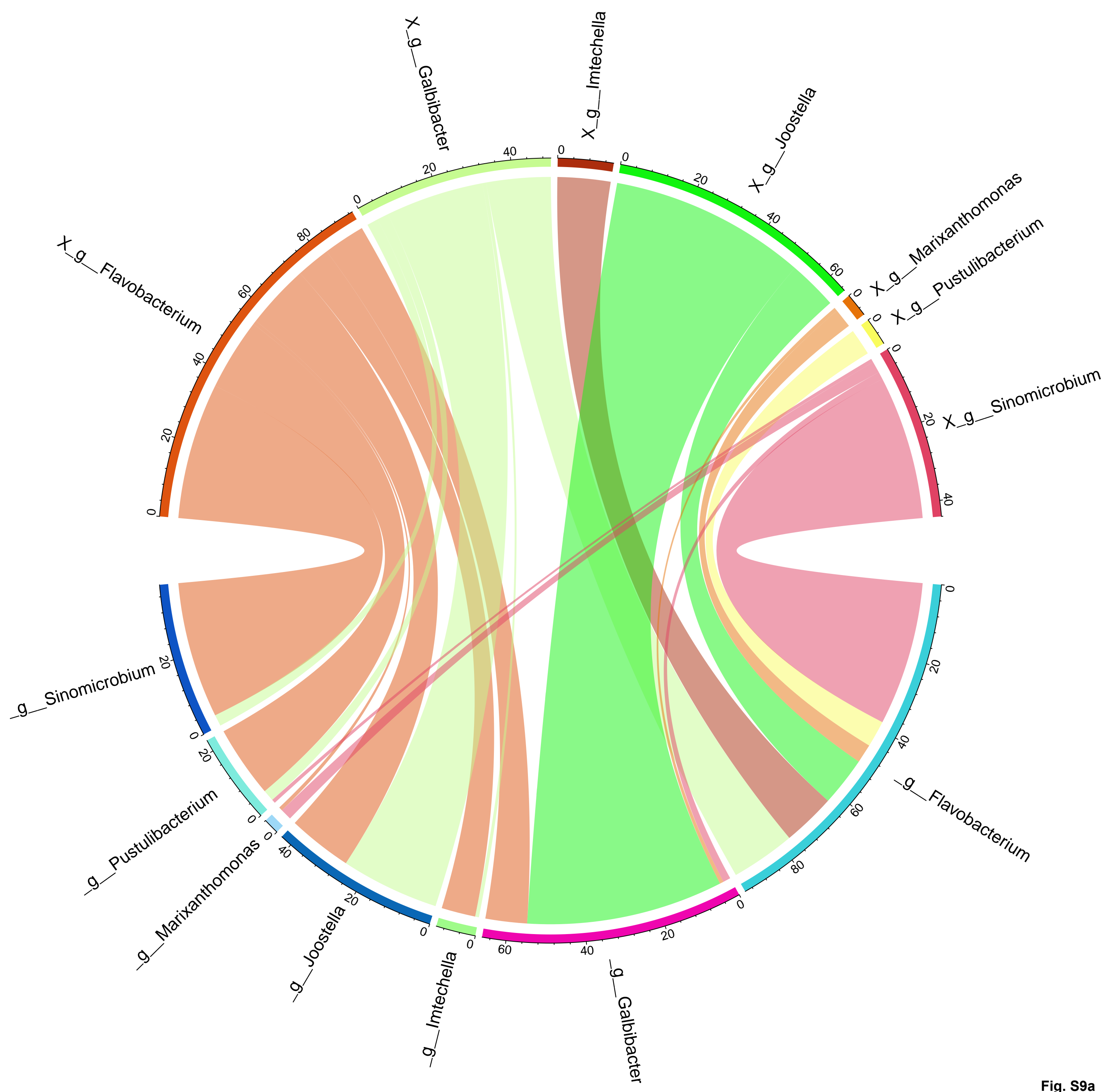

Fig. S9a

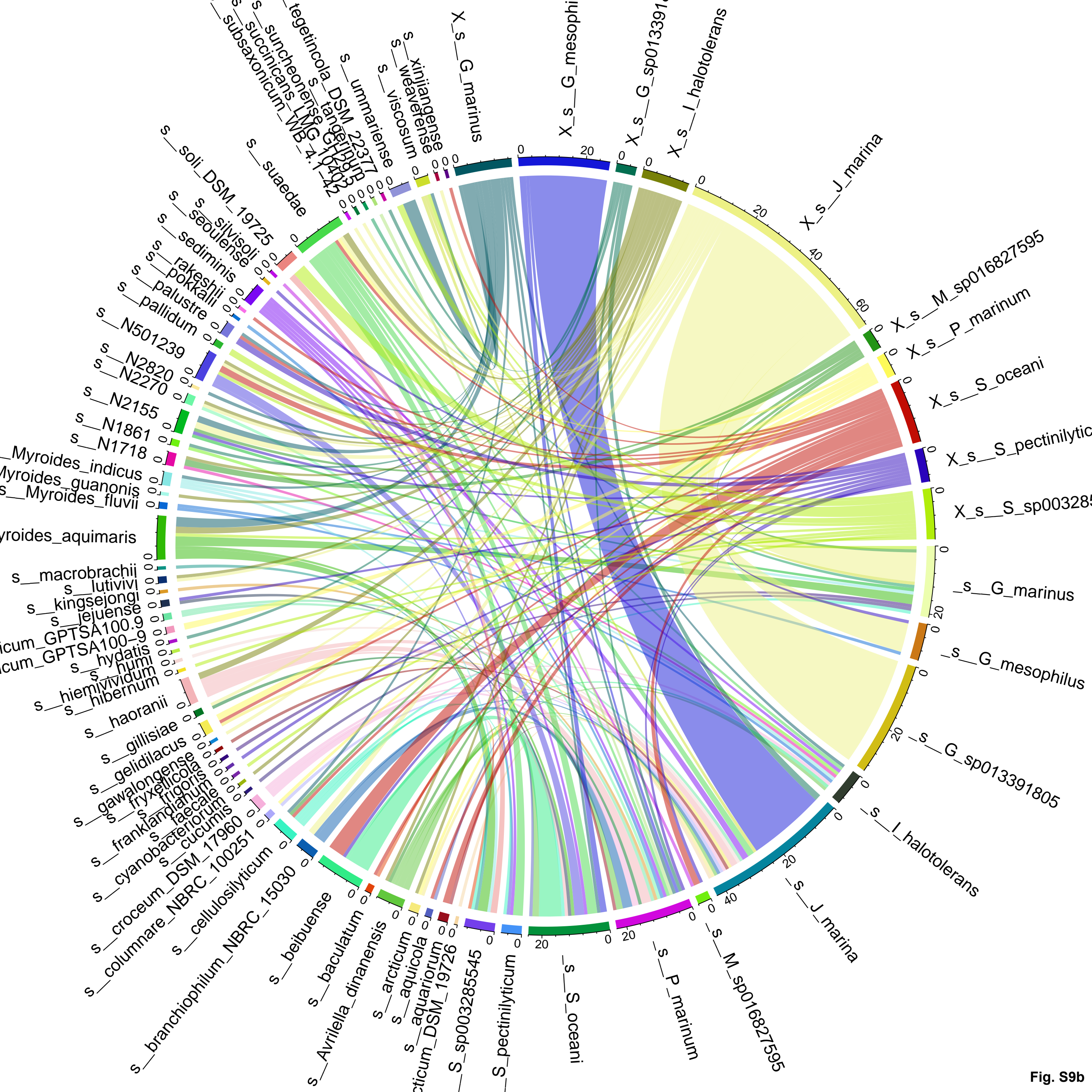

Fig. S9b

Supplement: Supplemental figures — Fig. S1 to S9. [file spectrum.01003-23-s0001.pdf]
