# Supplementary material for: Pangenome analysis provides insights into the genetic diversity, metabolic versatility, and evolution of the genus Flavobacterium
Source: Microbiol Spectr. 2023 Aug 18;11(5):e01003-23. doi: 10.1128/spectrum.01003-23 (PMC10655711; doi:10.1128/spectrum.01003-23)
Supplement: Supplemental legends — Legends for supplemental tables and figures. [file spectrum.01003-23-s0002.docx]

**Supplemental table and figure legends**

**Table S1.** Quality of full-length sequenced genomes, as determined using CheckM, for strains isolated in this study.

**Table S2.** Detailed information on reference genomes (from GTDB) included in this study and newly isolated strains.

**Table S3.** Summary statistics for the genomes analyzed in this study.

**Table S4.** Genes involved in carbohydrate assimilation in all *Flavobacterium* sp. genomes included in this study. BLASTp search against the Carbohydrate-active Enzymes Database (CAZy) annotated by the

three methods, HMMER, Diamond, and Hotpep, with the dbCAN database. DIAMOND: E-Value <1e-102, hits per query (-k) = 1, HMMER: E-Value <1e-15, coverage >0.35, Hotpep: Frequency >2.6, Hits >6. SignalP predicted by signal (v.4.1). GH, glycoside hydrolase; GT, glycosyltransferase; AA, Auxiliary Activities; CBM, carbohydrates-binding module; PL, polysaccharide lyase.

**Table S5.** Antibacterial biocide and metal resistance genes in 187 genomes analyzed by BacMed (http://bacmet.biomedicine.gu.se/).

**Table S6.** Horizontal gene transfer predicted by MetaCHIP (https://github.com/songweizhi/MetaCHIP).

**Figure S1.** Genome length (a) and GC% (b) represented by dot and frequency plots for all genomes classified based on isolation source. Marine, host-associated, and terrestrial groups are shown in light blue, light red, and light green, respectively.

**Figure S2.** Scatter plot showing the relationships between the genome length and number of the coding sequences (a), and coding density (b) for all genomes classified based on the isolation source. Dot plot of coding density (%) (c) in all genomes classified based on the isolation source. Significant difference in coding density (%) between host-associated and terrestrial groups presented by an estimation plot. Asterisk denotes *p* < 0.05. Marine, host-associated, and terrestrial groups are indicated by light blue, light red, and light green, respectively.

**Figure S3.** Heatmap of genome relatedness within members of the genus *Flavobacterium*, including newly isolated strains. Average nucleotide identity (ANI, a) and average amino acid identity (AAI, b) between pairs of genomes were calculated. The genomes of isolated strains are highlighted in boldface. Blue (marine), host-associated (magenta), and brown (terrestrial) denote their isolation source.

**Figure S4.** Pangenome features. Pangenome analyzed using Roary and plotted using Pagoo. (a) Number of conserved genes, (b) pangenome rarefaction curves, (c) gene frequency plots, and (d) pie chart with gene subsets (core, shell, and cloud; classified by Pagoo).

**Figure S5.** Pangenome analysis generated using Anvi’o for 20 *Flavobacterium* sp. genomes isolated in this study. The layers represent individual genomes organized by their phylogenomic relationships. Gene clusters were ordered according to a hierarchical clustering analysis of their presence/absence (inner dendrogram). Genomes were ordered based on the presence/absence of gene clusters. ANI values for genome comparisons are represented on a heatmap showing high similarity (red) and low similarity (white).

**Figure S6.** Distribution of Clusters of Orthologous Groups (COGs) in functional classes of all genomes classified by isolation source (a). Values presented by stacked frequency (%) of genes per COG category, and *x*-axis represents the isolation source. COG class U (intracellular trafficking, secretion, and vesicular transport) presented by dot and estimation plots (b). * and *** denotes *p* = 0.0190 and 0.0008, respectively.

**Figure S7.** Dot plot of tRNA (a) and rRNA (b) ratios among total predicted genes in all genomes classified based on the isolation source. Significant difference in tRNAs between host-associated and terrestrial groups presented by an estimation plot. Asterisk denotes *p* < 0.05.

**Figure S8.** Dot plot showing the ratio of carbohydrate-active enzymes (CAZy) to total predicted genes in all genomes classified based on the isolation source. Significant difference between marine and terrestrial groups. Asterisk denotes *p* < 0.0001.

**Figure S9.** Predicted gene flow of horizontal gene transfer events between genera (a) and species (b). Bands connect donors and recipients, with the width of the band proportional to the number of HGTs and the color corresponding to the donor.
